# Comparative genomics to explore phylogenetic relationship, cryptic sexual potential and host specificity of *Rhynchosporium* species on grasses

**DOI:** 10.1186/s12864-016-3299-5

**Published:** 2016-11-22

**Authors:** Daniel Penselin, Martin Münsterkötter, Susanne Kirsten, Marius Felder, Stefan Taudien, Matthias Platzer, Kevin Ashelford, Konrad H. Paskiewicz, Richard J. Harrison, David J. Hughes, Thomas Wolf, Ekaterina Shelest, Jenny Graap, Jan Hoffmann, Claudia Wenzel, Nadine Wöltje, Kevin M. King, Bruce D. L. Fitt, Ulrich Güldener, Anna Avrova, Wolfgang Knogge

**Affiliations:** 1 Department of Stress and Developmental Biology, Leibniz Institute of Plant Biochemistry, Halle/Saale, Germany; 2 Institute of Bioinformatics and Systems Biology, Helmholtz Zentrum München, Neuherberg, Germany; 3 Genomic Analysis, Leibniz Institute on Aging, Fritz Lipmann Institute, Jena, Germany; 4 Institute of Medical Genetics, Cardiff University, Cardiff, UK; 5 Exeter Sequencing Service, Biosciences, University of Exeter, Exeter, UK; 6 NIAB EMR, East Malling, UK; 7 Applied Bioinformatics, Rothamsted Research, Harpenden, Hertfordshire UK; 8 Systems Biology and Bioinformatics, Leibniz Institute for Natural Product Research and Infection Biology, Hans Knöll Institute, Jena, Germany; 9 Biological Chemistry and Crop Protection, Rothamsted Research, Harpenden, Hertfordshire UK; 10 Biological and Environmental Sciences, University of Hertfordshire, Hatfield, Hertfordshire UK; 11 Department of Genome-Oriented Bioinformatics, Technische Universität München, Wissenschaftszentrum Weihenstephan, Freising, Germany; 12 Cell and Molecular Sciences, The James Hutton Institute, Invergowrie, Dundee, Scotland; 13 Present address: Food Quality and Nutrition, Agroscope, Bern, Switzerland

**Keywords:** CAZymes, Effectors, Host specificity, *Leotiomycetes*, Non-ribosomal peptide synthetases, Phylogenetic evolution, Polyketide synthases, *Rhynchosporium*, Sex-related genes, Whole genome sequencing

## Abstract

**Background:**

The *Rhynchosporium* species complex consists of hemibiotrophic fungal pathogens specialized to different sweet grass species including the cereal crops barley and rye. A sexual stage has not been described, but several lines of evidence suggest the occurrence of sexual reproduction. Therefore, a comparative genomics approach was carried out to disclose the evolutionary relationship of the species and to identify genes demonstrating the potential for a sexual cycle. Furthermore, due to the evolutionary very young age of the five species currently known, this genus appears to be well-suited to address the question at the molecular level of how pathogenic fungi adapt to their hosts.

**Results:**

The genomes of the different *Rhynchosporium* species were sequenced, assembled and annotated using *ab initio* gene predictors trained on several fungal genomes as well as on *Rhynchosporium* expressed sequence tags. Structures of the rDNA regions and genome-wide single nucleotide polymorphisms provided a hypothesis for intra-genus evolution. Homology screening detected core meiotic genes along with most genes crucial for sexual recombination in ascomycete fungi. In addition, a large number of cell wall-degrading enzymes that is characteristic for hemibiotrophic and necrotrophic fungi infecting monocotyledonous hosts were found. Furthermore, the *Rhynchosporium* genomes carry a repertoire of genes coding for polyketide synthases and non-ribosomal peptide synthetases. Several of these genes are missing from the genome of the closest sequenced relative, the poplar pathogen *Marssonina brunnea*, and are possibly involved in adaptation to the grass hosts. Most importantly, six species-specific genes coding for protein effectors were identified in *R. commune*. Their deletion yielded mutants that grew more vigorously *in planta* than the wild type.

**Conclusion:**

Both cryptic sexuality and secondary metabolites may have contributed to host adaptation. Most importantly, however, the growth-retarding activity of the species-specific effectors suggests that host adaptation of *R. commune* aims at extending the biotrophic stage at the expense of the necrotrophic stage of pathogenesis. Like other apoplastic fungi *Rhynchosporium* colonizes the intercellular matrix of host leaves relatively slowly without causing symptoms, reminiscent of the development of endophytic fungi. *Rhynchosporium* may therefore become an object for studying the mutualism-parasitism transition.

**Electronic supplementary material:**

The online version of this article (doi:10.1186/s12864-016-3299-5) contains supplementary material, which is available to authorized users.

## Background

The genus *Rhynchosporium* (order: *Leotiales*) consists of haploid fungi that are pathogenic to grass species (*Poaceae*) including cereal crops. In an early review article [1], only two species were described, *R. secalis* and *R. orthosporum*, differing both in the shape of their two-celled conidia and in their host specificities. The name of the fungal genus originates from the beak (Greek: *rhynchos*) carried by one of the spore cells of *R. secalis*. In contrast, *R. orthosporum* produces cylindrical spores (Greek: *ortho*, straight). *R. secalis* was first isolated from rye (*Secale cereale*; [2]; cited in [1, 3]). Nevertheless, the major host worldwide is barley (*Hordeum vulgare*), on which the fungus causes the economically important disease leaf blotch or scald. *R. orthosporum* is a pathogen of orchard grass (cocksfoot, *Dactylis glomerata*).

It is usually assumed that plant pathogens originated on direct ancestors of their modern crop hosts and coevolved with them during domestication [4]. Therefore the center of origin of a pathogen is usually also the center of genetic diversity [5]. Gene diversity analysis of *Rhynchosporium* suggests, however, that the fungus did not originate from the Fertile Crescent [5], where its host plants barley and rye were domesticated about 10,000 years ago [6]. Instead, the genetic history of the fungus as it was reconstructed using the nucleotide sequence variation of the effector gene *NIP1* [7, 8] along with sequences from neutral RFLP loci [9] and microsatellite markers [10] suggests that *Rhynchosporium* emerged in Northern Europe following a host switch from a wild grass onto cultivated barley and rye after arrival of these plants about 5000–3000 years ago. From there the fungus spread globally most likely due to anthropogenic dispersal of infected host material [9–11], reaching regions such as South Africa, California, Australia and New Zealand only during the last few hundred years [10].

The occurrence of host-specialized ‘races’ of *R. secalis* was reported early on [1]. Nevertheless, for a long time there were conflicting reports about the host range of *Rhynchosporium* isolates [12]. Recently, multigene-sequencing and phylogenetic analysis (genealogical concordance phylogenetic species recognition; [13, 14]) demonstrated that *R. secalis* was actually a complex of at least three closely related host-specialized species [15]. Given that the fungus was initially described on rye, the name *R. secalis* was retained for the species infecting rye and triticale (x *Triticosecale* Wittmack). The species colonizing cultivated barley and other *Hordeum* spp. as well as brome grass (*Bromus diandrus*) was named *R. commune*, whereas *R. agropyri* is pathogenic to couch grass (*Elymus repens*; syn. *Agropyron repens*). Finally, fungal isolates from perennial ryegrass (*Lolium perenne*) were found to represent yet another species, *R. lolii* [16]. The cylindrical shape of the spores as well as phylogenetic analysis based on nucleic acid sequences position the latter species closer to *R. orthosporum* (cylindrical conidia group, CCG) than to the other three species (beaked conidia group, BCG). Due to its economic importance the majority of studies has been carried out on the barley-infecting fungal species, *R. commune* (in the older literature called *R. secalis*).

Phylogenetic concordance analyses based on the sequences of four housekeeping genes (ITS1-5.8S-rRNA-ITS2, EF1-α, α-tubulin, β-tubulin) and neutral RFLP loci using several hundred fungal isolates suggested that the CCG and the BCG branches separated 14,000–35,000 years ago, hence long before the advent of agriculture, with *R. lolii* diverging from *R. orthoporum* 4300–7200 years ago [16]. In contrast, *R. commune*, *R. secalis* and *R. agropyri* originated from a common ancestor only 1200–3600 years ago [8, 9, 16]. Furthermore, reconstructions of demographic growth indicate that populations of the three BCG species evolved in parallel. After the host shift, which was soon followed by the split of the three fungal lineages, they experienced a bottleneck due to their reproductive isolation. Specific adaptation to their modern hosts [15] led to the recovery of the sizes of all 3 populations in particular during the last 250 years [10, 17]. Thus, worldwide dissemination and population increase of the pathogen coincide with intensification and global expansion of cereal production [17, 18].

In evolutionary terms, the *Rhynchosporium* BCG species are very young and closely related. Thus it can be expected that their genomes did not diverge substantially yet, except for those genes that are involved in adaptation to the different hosts and/or environments. Hence, they represent an excellent basis to address the question of host specialization by comparative genomics. Genes under diversifying selection may be uncovered through a fully unbiased approach by scanning the genomes of several isolates from the different species. Alternatively, crucial processes associated with host adaption of a pathogen and epitomized in the concept of (quantitative) virulence may be defined *a priori*. This would allow the identification of patterns of adaptive evolution such as species-specifically occurring genes or of gene family expansion.

What are the factors that contribute to fungal virulence? *Rhynchosporium* development *in planta* starts with germination of conidia on the surface of host leaves and the penetration of fungal hyphae through the cuticle, frequently without forming obvious appressoria [19, 20]. Then, a long latent period of a few days up to several months [21, 22] without any visible disease symptoms follows. During this stage fungal mycelia are established between the cuticle and the outer wall of epidermis cells. Finally, after formation of a dense subcuticular fungal stroma necrotic lesions occur. New spores are formed both during the latent period and in the lesion areas. Penetration as well as subcuticular proliferation in the host tissue require the secretion of hydrolytic enzymes to degrade the cuticle and cell wall structures and to release carbohydrate nutrients form the plant cell wall polysaccharides. In addition, the physiology of the host needs to be redirected towards tolerating and accommodating the pathogen. For this purpose (hemibiotrophic) fungal pathogens secrete small molecules (secondary metabolites; [23]) as well as effector proteins. Therefore, the genes coding for cell wall-degrading enzymes, key secondary biosynthetic enzymes or protein effectors are prime targets of diversification/diversifying selection during adaptive evolution.

Finally, at the base of any considerations regarding evolutionary processes is the mating system of an organism. For *Rhynchosporium*, no teleomorph has been identified to date despite the presence of mating-type (*MAT*) genes [24]. Nevertheless, ‘the signature of sex’ [25], i.e., mating type equilibrium in most populations at least of the BCG species and evidence for *MAT* gene expression [5, 26, 27], high genotype diversity, gametic equilibrium among neutral markers and low levels of clonality, was found in fungal populations worldwide [5, 9, 18, 28, 29]. These results suggest that sexual recombination occurs cryptically but regularly.

This paper presents a comparative analysis of the genomes of isolates from four of the five *Rhynchosporium* species described to date. The intra-genus evolution and the broader phylogenetic relationship to other fungal species are re-analyzed. Furthermore, the possibility of sexual recombination is assessed using a genomics approach by screening for the presence of sex-related genes known to be involved in meiosis and recombination in other organisms. Finally, genes that may be involved in controlling host specificity, including those coding for key enzymes of fungal secondary metabolism or effector proteins are identified.

## Results and discussion

### Genome statistics

Three isolates of *R. commune* and one isolate each of *R. secalis*, *R. agropyri*, *R. lolii* and *R. orthosporum* were sequenced using a whole-genome shotgun strategy [30] with the Roche 454 GS FLX and the Illumina GAIIx platforms. *De novo* assembly resulted in a total sequence length of 52–58 Mb. Overall sequencing and assembly statistics are presented in Table 1. The assembled genome of *R. commune*, isolate UK7, consisting of only 163 scaffolds was established as the reference genome. Gene models for *R. commune*, isolate UK7, *R. secalis* and *R. agropyri* were generated by three *de novo* prediction programs with different matrices. Completeness of genomic predictions was assessed by BLASTp comparison to a set of 246 highly conserved fungal single-copy protein-coding homologs (SCPCH, [31]) and to a set of 248 core genes from the core Eukaryotic genes mapping approach (CEGMA, [32]; Table 2). The predicted coding sequences comprise about 30–33% of the genomic sequences, the estimated gene numbers range from 10,254 to 13,674 with about 50% coding for proteins of unknown function. Repetitive DNA elements were identified and categorized. The total coverage of repetitive sequences is relatively high as compared to other fungi reaching 31% in *R. commune*, but lower than in the close relative *Marssonina brunnea* f.sp. *‘multigermtubi’* (see below; 42% [33]).

**Table 1:** Genome sequencing and assembly

Parameter	*R. commune* (UK7)				*R.c.* (AU2)	*R.commune* (13-13)		*R. secalis*		*R. agropyri*				*R. ortho-sporum*
Primary seq. data	Illumina PE (GAIIx)	Illumina PE (GAIIx)	Illumina MP (GAIIx)	454	Illumina PE (GAIIx)	Illumina PE (GAIIx)	454^a^	Illumina PE (GAIIx)	454	Illumina PE (GAIIx)	Illumina PE (GAIIx)	Illumina PE (HiSeq)	Illumina MP (HiSeq)	Illumina PE (GAIIx)
No. of reads	18 Mio.	35 Mio.	6.5 Mio	2.37 Mio	32.1 Mio.	25.23 Mio.	2.21 Mio	34.7 Mio.	1.86 Mio.	34 Mio.	104 Mio.	26 Mio.	24 Mio.	38 Mio.
Read length	2 × 36 bp	2 × 151 bp, 270 bp dist.	2 × 151 bp, 3 kb dist.	377 bp	2 × 101 bp	2 × 76 bp	366.5 bp	2 × 101 bp	410 bp	2 × 101 bp, 300 bp dist.	2 × 100 bp, 180 bp dist.	2 × 151 bp, 270 bp dist.	2 × 151 bp, 3 kb dist.	2 × 101 bp
Total length	1.29 Gb	9.7 Gb	1.4 Gb	894 Mb	6.48 Gb	1.92 Gb	810 Mb	7.0 Gb	746 Mb	6.9 Gb	20.8 Gb	7.8 Gb	7.3 Gb	7.6 Gb
Nominal seq. depth	26x	194x	29x	18x	130x	25x	15x	140x	15x	137x	420x	156x	146x	150x
Assembly software	ALLPATHS-LG + SSPACE				Velvet	Velvet + Newbler + Minimus2		Newbler		ALLPATHS-LG + SSPACE				Velvet
Total seq. length (LFI)	55.59 Mb				54.32 Mb	53.93 Mb		56.80 Mb		52.18 Mb				58.16 Mb

^a^ 0.5 run 20 kb PE library, 1.5 runs 0.8 kb library

**Table 2:** Genome characteristics

	*R. commune* UK7	*R. secalis*	*R. agropyri*	*R. ortho-sporum*	*M. brunnea*
Assembly statistics					
Total scaffold length (Mb)	55.59	56,80	52.18	58.16	51.95
N50 contig (kb)	49.6	47.5	61.6		
N50 scaffold (kb)	800.5	113.7	303.4	104.3	
Scaffolds/Chromosomes	163	2129	508	29183	89
GC-content (%)	42.33	43.91	42.9	41.1	42.72
- coding (%)	49.86	49.85	49.75		54.12
- non-coding (%)	39.09	40.87	39.52		38.12
Coding sequence					
Percent coding (%)	30.06	33.84	33.08		28.78
Average gene size (bp)	1367.7	1271.6	1269.4		1491
Average gene density (gene/kb)	0.22	0.27	0.26		0.19
Protein-coding genes	12212	13151	13674		10027
Exons	38363	39806	41720		33423
Average exon size	434.4	419.1	415.1		394.6
Exons/gene	3.1	3	3.1		3.9
tRNA genes	113 (29 mt^a^)	117 (30 mt^a^)	107 (29 mt^a^)		nd
Validation of prediction					
SCPCH (246)	246	246	246	246	
CEGMAS (248)	248	248	247	247	
Secretome					
Secreted proteins ≥300aa	711	690	724		509
Small secreted proteins <300aa	457	574	655		429
Non-classical secreted proteins ≥300aa	77	90	89		61
Small non-classical secr. proteins <300aa	63	167	196		63
non-secreted protein	10904	11630	12010		8965
All proteins	12212	13151	13674		10027
Non-coding sequence					
Introns	26151	26655	28046		24747
Introns/gene	2.6	2.5	2.5		2.9
Average intron length (base)	80.5	82.4	83.1		118.5
Average intergenic distance (bp)	3013	2319	2398		3398
Repeat sequences					
DNA Transposon	8.04%	4.27%	6.89%		7.64%
LINE	0.39%	0.97%	0.44%		1.56%
SINE	0.07%	0.11%	0.07%		0.15%
LTR Retrotransposon	17.06%	7.84%	10.56%		27.41%
Unclassified nonLTR-Retrotransposon	0.02%	0.03%	0.00%		0.07%
Unclassified Retrotransposon	2.56%	3.35%	2.69%		2.54%
Unclassfied	0.22%	0.60%	0.42%		1.36%
Total TE class	28.21%	16.68%	20.88%		40.33%
Simple sequence repeats	0.90%	1.03%	0.94%		2.57%
Total excl. Tandem repeats	29.16%	17.79%	21.89%		43.07%
Tandem repeats	8.27%	5.05%	7.38%		11.46%
Total repeat coverage	31.23%	20.09%	24.00%		45.64%

^a^ mitochondrial

### Phylogenetic relationship

**Fig. 1:**
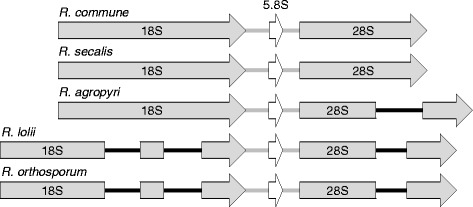
*Rhynchosporium* rDNA structures. The sequences of the rDNA regions of all *Rhynchosporium* species were obtained by Sanger sequencing. 18S, 5.8S and 28S genes are separated by ITS1 and ITS2, respectively (*grey lines*). The introns (*black lines*) in the 18S and 28S genes of *R. lolii* and *R. orthosporum* are identical except for 1 additional nucleotide in the *R. lolii* 28S intron. The 28S intron of *R. agropyri* is highly similar to those of the CCG species (87% identity) except for 5′- and 3′-terminal extensions of 99 and 87 bp, respectively

Phylogenetic analysis using multilocus DNA sequence data (concatenated α-tubulin, β-tubulin and ITS sequences) identified *R. secalis* as the closest sister species to *R. commune*, whereas the CCG species *R. orthosporum* is more distantly related [15, 17]. This relationship was supported in the present study by comparing the *Rhynchosporium* rDNA gene structures (Fig. 1). The genes of *R. commune* and *R. secalis* are highly similar lacking any introns, whereas an intron is present in the 28S gene of *R. agropyri*. The CCG species *R. orthosporum* and the recently described *R. lolii* [16] carry two introns of the same size in the 18S gene and single introns differing in length by one nucleotide in the 28S gene. The latter are homologous to the 28S intron of *R. agropyri* and located at the same site, but their sequences are 99 bp and 87 bp shorter at the 5′ and the 3′ side, respectively. In contrast to these results, phylogenetic analysis based on concatenated sequences of 13 core mitochondrial genes resulted in *R. agropyri* and *R. secalis* as being most closely related [30].

**Fig. 2:**
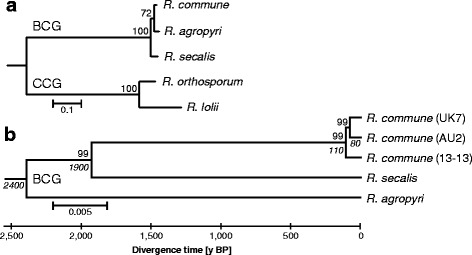
*Rhynchosporium* evolutionary relationships. **a** *Rhynchosporium* subtree of the *Leotiomycetes* phylogeny (*cf.* Additional file 1: Figure S1). The nucleotide sequences of 18S rDNA, 28S rDNA, ITS region, elongation factor EF1-a and RNA polymerase II subunits RPB1 and RPB2 were concatenated. **b** SNP-based phylogeny of the BCG species including the three *R. commune* isolates UK7, AU2 and 13-13. The evolutionary history was inferred using the Minimum Evolution method (optimal tree with sum of branch length = 1.022). The tree is drawn to scale. All nucleotide positions containing gaps and missing data were eliminated, leaving a final dataset of 5,904,161 positions. Scale: number of substitutions per site and SNPs per position, respectively. Bootstrap numbers are given above branching points, divergence times in italics below branching points. BCG, beaked conidia group; CCG, cylindrical conidia group

To better resolve the *Rhynchosporium* phylogeny a two-step analysis was carried out in the present study. The first step aimed at confirming the general position of the *Rhynchosporium* genus in the *Leotiomycetes* class of Ascomycota [34]. To this end, the sequences of three protein-coding genes (EF1-α, RNA polymerase II subunits RPB1 and RPB2) and of almost complete rDNA regions (18S rDNA, ITS1-5.8S rDNA-ITS2, 28S rDNA; [35]) from 12 members of the *Leotiomycetes* class were used in lack of complete genome sequences of these fungi other than *Botryotinia fuckeliana* [36]. The resulting phylogeny shows the *Rhynchosporium* BCG branch with *R. commune* and *R. agropyri* as the closest sister species alongside the CCG branch (Fig. 2a, Additional file 1: Figure S1). In a second step, to resolve the BCG evolution in more detail, single nucleotide polymorphisms (SNPs; Additional file 2: Table S1) were identified after aligning the genomes of BCG species and isolates (Fig. 2b). The phylogenetic tree inferred from the concatenated SNPs again confirms that *R. agropyri* branched off “shortly” before *R. commune* and *R. secalis* diverged. According to the published evolutionary time scale the former occurred 1200–3600 years ago [8, 9, 16], the latter about 500 years later. In contrast, the age of the 3 *R. commune* isolates is probably around 100 years or less.

**Fig. 3:**
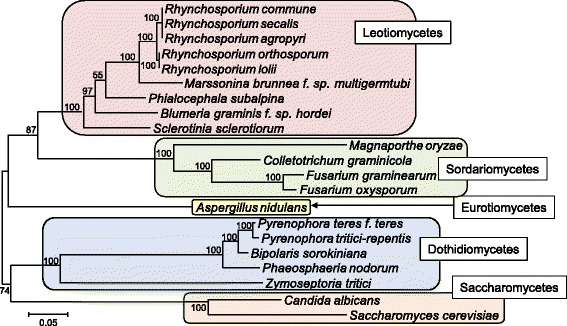
Integration of the genus *Rhynchosporium* into the fungal systematics. The concatenated amino acid sequences of elongation factor EF1-a and of the polymerase II subunits RPB1 and RPB2 from 21 taxa were used to construct the phylogenetic tree. Numerals on the nodes represent the percentages from 500 bootstraps. Scale: number of substitutions per nucleotide

Using the amino acid sequences of the three proteins EF1-α, RPB1 and RPB2 the relationship of *Rhynchosporium* to more distantly related plant-interacting fungal species was established (Fig. 3). The closest relative of *Rhynchosporium* spp., the poplar pathogen *M. brunnea* f.sp. *‘multigermtubi’* [33], belongs to the *Dermataceae*, a family of one of the most diverse ascomycete orders, the *Helotiales*. This confirms a previous ITS-based phylogenetic analysis, which located *R. commune* and *R. orthosporum* between the *Dermataceae* species *Tapesia yallundae* (syn. *Oculimacula yallundae*) and *Pyrenopeziza brassicae*, pathogens of grasses and oil seed rape (*Brassica napus*), respectively [34].

**Fig. 4:**
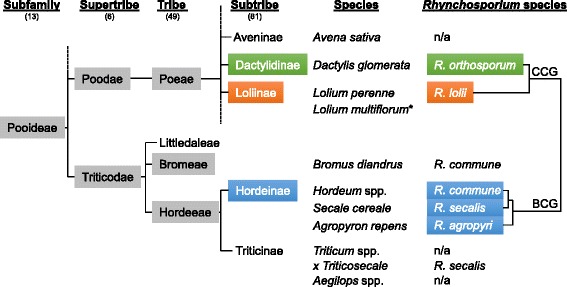
*Rhynchosporium* species and their hosts from the *Poaceae* family. The *Rhynchosporium* systematics is contrasted with the section of the grass systematics [37] containing all known host species. Numbers in brackets indicate the total number in this order. *Two *R. commune* isolates were described to be able to cross the *Poodae-Triticodae* border, being pathogenic to *Lolium multiflorum* and *Hordeum vulgare* [16]. BCG: beaked conidia group, CCG: cylindrical conidia group, n/a: not a host species

All host species of *Rhynchosporium* belong to the *Pooideae* subfamily of *Poaceae* [37, 38]. However, while the fungal BCG species, *R. commune*, *R. secalis* and *R. agropyri*, infect closely related grass species from genera of the subtribe *Hordeinae* (supertribe *Triticodae*, tribe *Hordeeae*), the CCG members, *R. lolii* and *R. orthosporum*, are pathogenic to species from the more distantly related subtribes *Loliinae* and *Dactylidinae* (supertribe *Poodae*; Fig. 4). Nevertheless there are exceptions to this rule. Firstly, from *Bromus diandrus*, a grass species belonging to the *Bromeae* tribe of the *Triticodae* supertribe, a few isolates were collected that grouped into the *R. commune* lineage [17]. Interestingly, in an early report isolates from brome grass were unable to infect other grass species tested including barley [1]. Secondly, triticale became susceptible to *Rhynchosporium* about 30 years after its introduction as a crop [39]. This cereal species originated from hybridizing the non-host species wheat and the host species rye and was assigned to the *Triticinae* subtribe of the *Hordeae* tribe (supertribe *Triticodae*). *Rhynchosporium* isolates collected from triticale grouped into the *R. secalis* lineage [17]. Lastly, two strains of *R. commune* were isolated from Italian ryegrass (*Lolium multiflorum*) that were also pathogenic to barley [16] and, thus, able to colonize hosts from both *Triticodae* and *Poodae* supertribes. Despite these exceptions, however, the relationship found between the *Rhynchosporium* species corresponds largely to host systematics.

### Genes related to sexual reproduction

**Fig. 5:**
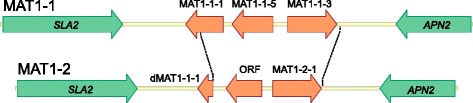
*Rhynchosporium* *MAT * gene loci. MAT1-1, *R. commune* UK7 (13,701 bp); MAT1-2, *R. agropyri* (12,801 bp). Dotted lines mark the idiomorphic regions. The genes flanking the MAT loci code for a cytoskeletal protein (SLA2) and a DNA lyase (APN2), respectively

Work since the 1930s has failed to demonstrate a sexual cycle in any *Rhynchosporium* species. Availability of the *Rhynchosporium* genomes allows now a genomic interrogation to assess whether the genetic machinery required for sexual reproduction is present or not. Such insights into the pathogen life cycle are important as this impacts on the possibility for recombination and evolution of the pathogen [40]. Despite the apparent absence of a teleomorph *R. commune* isolates have been shown to possess the mating type loci *MAT1-1* or *MAT1-2*, which are typical for heterothallic ascomycetes [24]. *MAT1-1* is characterized by genes coding for proteins with two different DNA-binding domains, an α-box motif in *MAT1-1-1* and a high-mobility group (HMG) DNA-binding motif in *MAT1-1-3*. Both loci were also found in the other BCG species, *R. secalis* and *R. agropyri*, whereas the CCG species *R. orthosporum* and *R. lolii* lack *MAT1-2* [27]. In the present study, only the *R. agropyri* isolate had the *MAT1-2* genotype, whereas all others carried the *MAT1-1* locus. Closer inspection of the *MAT1-1* locus revealed the presence of an additional ORF coding for a protein with about 30% similarity to the MAT1-1-5 protein from *B. cinerea* and several *Sclerotinia* species (Fig. 5). An additional ORF with homology to a hypothetical gene from the poplar pathogen *M. brunnea* f.sp. ‘*multigermtubi*’ was detected at the *MAT1-2* locus. However, this ORF codes for a protein with no homology to a functional protein from other organisms. Sequence comparison of the *MAT* loci demonstrated that the idiomorphic region comprises most of the *MAT1-1-1* gene and the genes *MAT1-1-5* and *MAT1-1-3* in the *MAT1-1* locus (*c*. 4150 bp). In *MAT1-2* it spans from the truncated *MAT1-1-1* gene to the 3′ end of the *MAT1-2-1* gene (*c*. 3600 bp). Both *MAT* loci are flanked by the genes *SLA2* and *APN2* coding for a cytoskeletal protein and a DNA lyase, respectively. The positions of these genes are highly conserved across *Pezizomycotina* species [41–44].

Meiosis is “at the heart of sexual reproduction” of Eukaryotes [45]. Therefore, searching for genes involved in meiosis and sexual reproduction can identify the capacity for cryptic sexuality. To this end, the *Rhynchosporium* genomes were screened using a “meiosis detection tool kit” [46], which comprises a set of genes defining a “core meiotic recombination machinery” (Table 3, *cf.* Additional file 3: Table S2A) [47, 48]. The products of these genes are involved in generating meiotic double-strand breaks, crossing over and cohesion of sister chromatids and homologous chromosomes. Bidirectional BLASTp analysis detected 26 out of the 29 core meiotic genes in the *Rhynchosporium* (with the exception of *R. lolii*) genomes with sequence similarities mostly exceeding 50%. For two additional proteins, HOP2 and MND1, the similarity to *Rhynchosporium* proteins is low (*c*. 30%), although the functional domains were identified. Many genes required for meiosis also have a role in mitosis. However, nine meiosis-specific genes are regarded to reliably predict the capacity of a sexual cycle [48]. Three of these genes that code for elements of the synaptonemal complex, HOP1, HOP2 and MND1, are missing in *Rhynchosporium* (*HOP1*) or their products show only low similarity with *Rhynchosporium* proteins (HOP2, MND1). Nevertheless this does not argue against sexual reproduction, because these genes are also absent from the genomes of several fungi with known sexuality (*Neurospora crassa*, *Gibberella zeae*, *Magnaporthe grisea*, *Ustilago maydis*) and of other sexual organisms such as *Drosophila melanogaster* [48]. Hence, meiosis appears to be possible without these gene products. Transcripts for all but two (*DMC1*, *MSH5*) of the 26 core meiotic genes have been identified in germinated conidia and/or cDNA libraries from infected barley leaves (Table 3). This confirms that the core meiotic genes are not only present in the *Rhynchosporium* genomes but are actively transcribed.

**Table 3:**
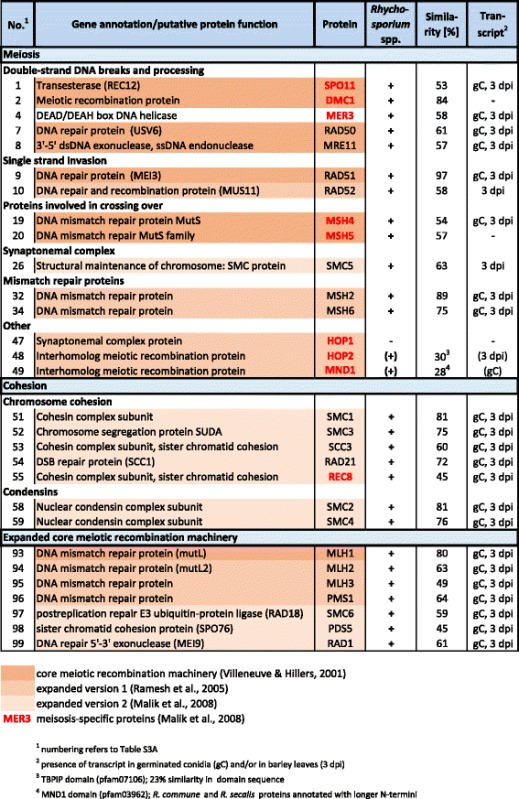
Core meiotic genes

Of an additional set of 70 genes involved in different aspects of meiosis and recombination in *N. crassa*, *Saccharomyces cerevisiae* and *Podospora anserina*, 68 were found to be maintained in the genomes of *R. commune*, *R. agropyri* and *R. orthosporum* (Additional file 3: Table S2A). Two genes, *KEX1* and *RAM1*, were missing only from the genome of *R. secalis*. Transcripts for 63 of these genes, including those coding for meiotic recombination protein REC4 and all pheromone proteins essential for mating, have been identified in germinated conidia and/or cDNA libraries from infected barley leaves. Finally, 74 genes known to be involved in different aspects of sexual reproduction in the aspergilli such as sensing of environmental signals that control sexual cycle induction, intracellular signal transduction, transcription control and ascospore production were examined (Additional file 3: Table S2B; [49]). Outside the *MAT* genes and the genes coding for pheromones and their receptors, only 5 genes were not found in the *Rhynchosporium* genomes, whereas for 12 gene products a relatively weak similarity with the *A. nidulans* proteins (<40%, 4 with <30%) was observed. Transcripts for 64 of the 65 genes, including *MAT1* and *esdC*, have been identified in germinated conidia and/or cDNA libraries from infected barley leaves. Taken together these results provide strong additional evidence for the presence and expression of the genetic machinery required for sexual reproduction to occur in the *Rhynchosporium* life cycle.

### Enzymes involved in cell wall degradation

Plant cell walls are built up of polysaccharides (cellulose, hemicellulose, pectin), (glyco-) proteins and aromatic polymers (lignin). Phytopathogenic fungi need to degrade this barrier to penetrate and colonize host tissues as well as to release nutrients for colony growth. To this end, they produce many carbohydrate-active enzymes (CAZymes) that cleave or modify oligo- and polysaccharides as well as other glycoconjugates. A recent genome comparison comprising 103 fungal species from different classes revealed a great diversity in the cell wall-degrading capacity [50]. In phytopathogens, CAZyme numbers correlate by and large with the fungal life style. In both hemibiotrophic and necrotrophic fungi large numbers of CAZymes are found, whereas obligately biotrophic fungi such as *Blumeria graminis* have smaller numbers. Furthermore, polygalacturonases of the GH28 family have been found to be less abundant in monocot- as compared to dicot-infecting fungi. When the CAZy database [51, 52] was used to annotate the *Rhynchosporium* BCG genomes, *R. commune*, *R. secalis* and *R. agropyri* had CAZyme numbers characteristic for monocot-infecting hemibiotrophic and necrotrophic fungi [50] (Additional file 4: Figure S2, Additional file 5: Table S3).

**Fig. 6:**
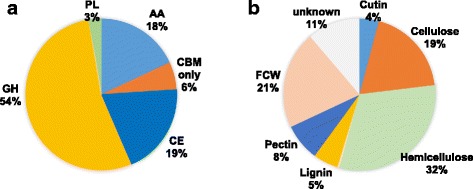
Cell wall-degrading enzymes of *R. commune*. **a** Genes coding for secreted CAZyme. AA, auxiliary activities, CBM only, proteins with carbohydrate-binding modules but lacking known enzyme activities, CE, carbohydrate esterases, GH, glycoside hydrolases, PL, polysaccharides lyases. **b** Genes coding for enzymes that target the different cell wall components

To reach their substrates, the cell wall-degrading enzymes (CWDEs) need to be secreted to the apoplast. In addition to the classical signal peptide-based secretion pathway through the ER/Golgi system, mechanisms independent of this system exist in Eukaryotes for unconventional secretion of proteins lacking a signal peptide [53, 54]. A sequence-based prediction method, SecretomeP, has been developed that is capable of identifying proteins secreted through the latter pathway [55]. Therefore, the *Rhynchosporium* CAZymes were tested for the presence of a classical N-terminal secretion signal and for the likelihood of unconventional secretion. Furthermore, enzyme numbers were corrected for modular enzymes, which are characterized by more than one annotation. In most cases (28 out of 39 proteins), an enzyme domain was combined with one or more carbohydrate binding domains (CBMs). As a result, about 330 CWDEs were identified for each *Rhynchosporiu*m species (Table 4, Additional file 5: Table S3, Fig. 6a). They group into glycoside hydrolases (GH, *c.* 54%), carbohydrate esterases (CE, *c.* 19%), polysaccharide lyases (PL, *c.* 3%), and auxiliary redox enzymes involved in lignocellulose breakdown (AA, *c*. 18%).

**Table 4:** Cell wall degrading enzymes

CAZy family	*R. commune*		*R. secalis*		*R. agropyri*	
	Number	[%]	Number	[%]	Number	[%]
GH^a^	182	53.8	176	53.7	183	54.3
CE^b^	66	19.5	62	18.9	64	19.0
PL^c^	9	2.7	9	2.7	9	2.7
CBM^d^ only	20	5.9	20	6.1	20	5.9
AA^e^	61	18.0	61	18.6	61	18.1
Ʃ	338	99.9	328	100.0	337	100.0
CWDE substrate	*R. commune*		*R. secalis*		*R. agropyri*	
	Number	[%]	Number	[%]	Number	[%]
Cutin	14	4.1	14	4.3	14	4.2
Cellulose	64	18.9	64	19.5	64	19.0
Hemicellulose	107	31.7	101	30.8	108	32.0
Lignin	18	5.3	18	5.5	18	5.3
Pectin	27	8.0	27	8.2	27	8.0
FCW^f^	70	20.7	68	20.7	70	20.8
unknown	38	11.2	36	11.0	36	10.7
Ʃ	338	99.9	328	100.0	337	100.0

^a^glycoside hydrolases

^b^carbohydrate esterases

^c^polysaccharide lyases

^d^carbohydrate-binding modules

^e^auxiliary activities

^f^fungal cell wall

In addition to enzymes, proteins possessing one or more carbohydrate-binding modules but lacking domains for enzyme activity were found (CBM only, *c*. 6%). Among these the LysM motif (CBM50) has attracted most attention [56]. This module has general N-acetylglucosamine binding properties and is therefore able to bind to chitin or chitin-like carbohydrates [57]. In fungi, LysM domains are predominantly found in secreted LysM effector proteins and in subgroup C chitinases. In the *Rhynchosporium* genomes seven proteins are encoded that contain one or more LysM domains (LysM type A [57]; LysM1, LysM5 and LysM7 with one domain, LysM2 with two domains, LysM3 with three domains, LysM4 and LysM6 with four domains). In addition, two genes code for enzymes that contain LysM domain pairs (Additional file 6: Table S4A), a subgroup C chitinase (CAZy family GH18; LysM type B; see below), which also possesses a different chitin-binding motif (CBM18), and a putative peptidoglycan lytic transglycosidase (CAZy family GH23). Four of the LysM proteins are secreted through the classical pathway, whereas three of the proteins, LysM5, LysM6 and LysM7 and the GH23 enzyme may be secreted via the unconventional pathway. A central transmembrane domain predicted for LysM7 may cause this protein to reside in the plasma membrane with the LysM domain oriented outwards. Interestingly, in *M. brunnea* a family of 24 genes was identified that code for effector proteins of 142–151 aa with 1 or 2 LysM motifs [58]. This family is completely missing in *Rhynchosporium*. Two representative members of this gene family were shown to weaken the chitin-induced defense response when expressed in *Arabidopsis thaliana*. As proposed for the effector proteins ECP6 from *Cladosporium fulvum* [59], Mg3LysM from *Mycosphaerella graminicola* (*Zymoseptoria tritici*; [60]) and Slp1 from *Magnaporthe oryzae* [61], this effect is probably due to the sequestration of chitin oligosaccharides that occur as breakdown products of fungal cell walls during invasion and that are able to trigger host immunity. Other LysM effectors including Avr4 from *C. fulvum* [62], Mg1LysM and Spl1 prevented hyphal lysis by plant chitinases [60, 61].

When the *Rhynchosporium* CWDEs were assigned to their putative substrates (cellulose, hemicellulose, pectin, lignin, other polysaccharides), about 21% were found to act or are likely to act on the fungal cell wall, whereas *c*. 64% presumably target the host cell wall (Table 4, Additional file 5: Table S3, Fig. 6b). For about 11% of the enzymes their substrate is unknown or ambiguous. Fourteen enzymes (4%) are putative cutinases. These enzymes, albeit no proper CWDEs, are required at a very early stage of pathogenesis to allow or facilitate the fungal penetration of the plant surface.

The physiological role of the CAZymes targeting the fungal cell wall is thought to be in cell wall remodeling during fungal morphogenesis (septum formation, hyphal branching, spore germination). The cell walls of filamentous fungi are mainly built up of β-1,3-glucan (65–90%), glycoproteins (20–30%) and chitin (10–20%; [63]). Accordingly, glucan-degrading enzymes constitute the biggest group of hydrolytic enzymes encoded in the *Rhynchosporium* genomes with at least 22 members (CAZy families GH17, GH55, GH81, GH128, GH72, possibly supplemented by members of families GH3, GH16 and GH131 [64]). Eight enzymes presumably target glycoproteins (GH20, GH114, GH125; Additional file 6: Table S4). In addition, 17 chitinolytic enzymes were identified (GH18, GH75, GH89; CE4; Additional file 6: Table S4). Eight of the 14 chitinases (GH18) belong to subgroup A (40–50 kDa, no CBMs; [65]). The biggest of these chitinases is predicted for unconventional secretion. One member of this subgroup was found to lack a signal peptide and, hence, to be located intracellularly (*cf.* [66]). Three chitinases belong to subgroup B (30–90 kDa, CBMs), but two additional enzymes may fall into this group despite their high molecular mass (c. 120 kDa). Subgroup C chitinases were defined to have a high molecular mass (140–170 kDa) and to carry a chitin-binding domain (CBM18) and LysM motifs. These criteria are fulfilled by one *Rhynchosporium* enzyme of almost 150 kDa. This enzyme may not be fully released into the apoplast, but rather tethered to the outside of the plasma membrane as suggested by a predicted C-terminal transmembrane helix.

In *Trichoderma atroviride* the gene coding for subgroup C chitinase TAC6 is located next to the gene encoding the TAL6 protein, which contains 7 LysM motifs and was considered to inhibit spore germination [67]. TAC6 appears to be inactivated by a deletion in the catalytic domain and hence to be a chitin-binding protein. Interestingly, a *tac6* deletion mutant was reported to grow faster than the wild type, suggesting a growth decelerating function of the protein and thus a function in the regulation of fungal development. Similar to *T. atroviride*, the subgroup C chitinase-encoding gene of *R. commune* is located next to a gene coding for a protein with 4 LysM motifs, LysM4. Furthermore, *chitinase-LysM* gene pairs are also found for both high molecular mass subgroup B chitinases. Their genes are located next to the genes coding for LysM1 and for a protein that lacks a LysM domain but has high similarity to a LysM protein from *Zymoseptoria brevis* [68], respectively. This *chitinase-LysM* gene arrangement may therefore point to the participation of LysM proteins in a more wide-spread mechanism to control fungal growth *in planta*.

Regrouping of the plant CWDEs according to the different types of cell wall substrates suggested that *c*. 32% are involved in hemicellulose degradation and about 19% target cellulose (Fig. 6b). The enzymes acting on pectin (8%) and lignin (5%) constitute smaller groups, thus reflecting the low content of these materials in the cell walls of grasses. Due to their large number and the functional redundancy of enzyme activities involved in cell wall degradation, the likelihood is generally presumed to be small that any one of these enzymes is responsible for host specificity. Recently, however, a combination of comparative genomics and transcriptomics revealed that members of CWDE families from *Z. tritici* are differentially transcribed during pathogenesis [69]. This suggested the specific occurrence of at least some CWDEs during relevant stages of the fungal life cycle rather than their general functional redundancy. In addition, selection analysis yielded evidence that a few cutinases, cellulases and hemicellulases may be involved in host adaptation or evasion of host recognition.

Based on the genome sequences, a very coarse intra-genus comparison was done for the *Rhynchosporium* CAZymes, which considered the presence or absence of genes in a family. In several of the CAZy families (e.g., arabinose family GH93, poorly characterized family GH89 [70]) single members are missing from one of the three BCG species. It remains to be shown however whether these differences reflect differences in the composition of the targeted cell walls and, thus, adaptations to the host.

### Secondary metabolism

Generally, secondary metabolites can be regarded as tools that play a role in the interaction of the producing organism with its environment. Fungal secondary products are not only notorious toxins, but also mediators of chemical communication with other microbes and the environment or defense agents [71]. Hence, they also play a more or less prominent role in the interaction of phytopathogenic fungi with their host plants. Most fungal secondary metabolites belong to one of four structural classes, polyketides, non-ribosomal peptides, cyclic terpenes or tryptophan-derived indole alkaloids [72]. Among the fungal compounds with known effect in plant-pathogen interactions are several of the host-specific toxins of the *Dothideomycetes* (for review s. [73]). T-toxin from *Cochliobolus heterostrophus* and PM-toxin from *Mycosphaerella zeae-maydis* are linear polyketides. Victorin from *Cochliobolus victoriae*, AM-toxin from *Alternaria alternata* and HC-toxin from *Cochliobolus carbonum* are cyclic non-ribosomal peptides, whereas HS-toxin from *Bipolaris sacchari* is a sesquiterpene galactoside. Several fungi produce perylenequinone toxins (e.g. cercosporin from *Cercospora* spp.). These are cyclic polyketides with non-host-specific activity. Some secondary metabolites have a less pronounced effect in the interaction with host plants. Examples are the polyketide botcinic acid and the sesquiterpene botrydial, which play a redundant role in the virulence of *Botrytis cinerea* on bean leaves [74]. Finally, many secondary products have an impact on human and livestock health; these include the ergot indole alkaloids with their tryptophan-derived lysergic acid moiety [75] or the sesquiterpenoid trichothecenes [76].

The only secondary metabolite described to date from a *Rhynchosporium* species is orthosporin, a polyketide from *R. orthosporum* [77]. This isocoumarin was also isolated from *Drechslera siccans*, a pathogen of oats (*Avena sativa*), perennial ryegrass (*L. perenne*) and Italian ryegrass (*L. multiflorum*). It is phytotoxic causing necrosis on leaves of several plants, but not on perennial ryegrass [78], and it has an inhibitory effect on root growth of the host plant, orchard grass (*D. glomerata*) and lettuce (*Lactuca sativa*).

The four major fungal secondary metabolite classes are synthesized via four characteristic key enzymes [72, 79], polyketide synthases (PKS), non-ribosomal peptide synthetases (NRPS), terpene cyclases (TC) or dimethylallyl tryptophane synthases (DMATS). In addition, several hybrid compounds are known, which are either synthesized by PKS-NRPS hybrid enzymes or by two separate enzymes that are encoded in a single gene cluster. The *Rhynchosporium* genomes were screened for the presence of these key enzyme genes to find clues for their possible involvement in determining host specificity. In almost all isolates/species three *DMATS* genes and one *TC* gene were identified, whereas *DMATS3* was missing only in *R. orthosporum*, suggesting that a host-specific role of these enzymes is unlikely.

*PKS* genes constitute the largest group of key enzyme genes of secondary metabolism. PKS catalyze the synthesis of a large and structurally diverse group of chemical substances from acyl-CoA precursors. Fungal PKS are usually multidomain enzymes that elongate and modify their products iteratively (type I PKS; [80]). Three domains are essential for polyketide synthesis, ketoacyl CoA synthase (KS), acyltransferase (AT) and a phosphopantetheine attachment site (PP). Several other enzymatic motifs such as the three β-keto processing domains ketoreductase (KR), dehydratase (DH) and enoyl reductase (ER) and a methyl transferase domain (MT) are not found in all PKS. Lack of all or some of the reducing domains yields non-reducing or partially reducing PKS. The polyketide product is usually released from the enzyme through thioreductase (TR), thioesterase (TE) or transferase activities. Twelve *PKS* genes (*PKS1-12*) including 2 *PKS-NRPS* genes (*PKS1*, *PKS11*) occur in all BCG species (Table 5). In contrast, *PKS13* was not found outside *R. commune* isolates and appears to be species-specific, whereas an additional gene, *PKS14*, was present only in the genome of *R. commune* isolate AU2. Orthologs of eight of the *PKS* genes were also identified in *R. orthosporum* along with four unique genes including two *PKS-NRPS* hybrid genes (*PKS15*, *PKS16*). In the closely related *M. brunnea*, orthologs of only 6 *Rhynchosporium* genes were identified along with two unique *PKS* genes. As the conclusion, the specific occurrence of several of the genes may suggest their involvement in the biosynthesis of polyketides with a role in interaction specificity at the species and isolate level.

**Table 5:**
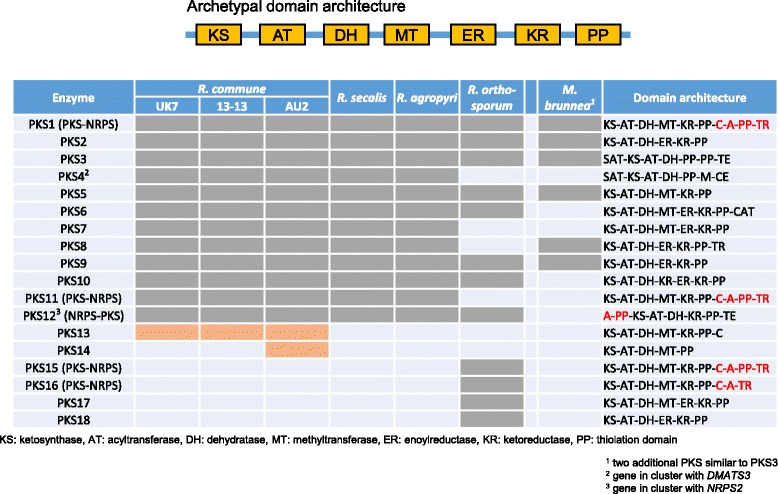
Polyketide synthases

**Fig. 7:**
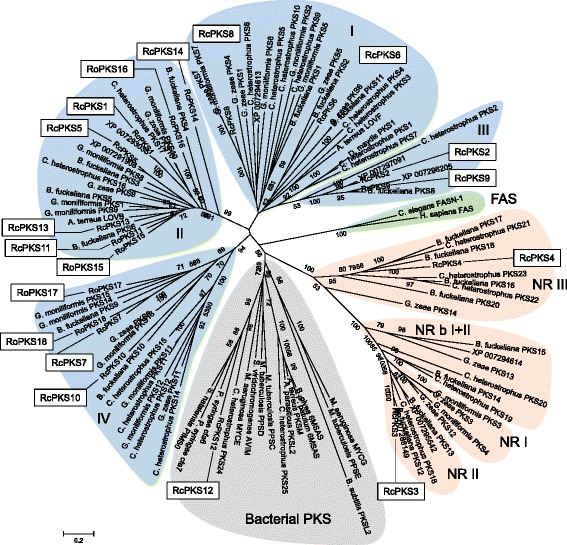
PKS phylogenetic tree. The amino acid sequence of the KS domains from 114 fungal and bacterial PKS were used to construct the phylogenetic tree. Numerals on the nodes represent the percentages from 500 bootstraps. Numerals <50 were omitted. Scale: number of substitutions per nucleotide. Colored backgrounds indicate enzyme groups: *blue*, reducing PKS clades I-IV; *orange*, non-reducing PKS clades NRI-III and NR bI + II [81]; *grey*, bacterial PKS; *green*, fatty acid synthases (FAS)

The phylogeny of type I PKS from ascomycetes and bacteria as inferred from KS domain relationship revealed three main clades comprising fungal reducing PKSs, fungal non-reducing (NR) PKSs and bacterial PKSs [81]. The fungal clades are further subdivided into four reducing subclades (I-IV), three NR subclades (NR I-III) and a subclade basal to NR subclades I and II (NR b I + II). In addition, the bacterial PKS clade contains two subclades of fungal PKS. This phylogeny was first reconstructed based on the KS domain sequences from 86 ascomycete PKS (including eight from *M. brunnea* [33]), ten bacterial PKS and two animal fatty acid synthetases. Subsequently, it was complemented with the KS domain sequences of the 18 *Rhynchosporium* PKS (Fig. 7), which were predicted by BLASTp analysis [82] and comparison to the PFAM 28.0 database (Table 5; [83]). The archetypal PKS domain architecture KS-AT-DH-MT-ER-KR-PP [81] was found in only three *Rhynchosporium* PKS, RcPKS6, RcPKS7 and RoPKS17 (Table 5), which are members of reducing subclades I and IV, respectively. Both subclades contain additional *Rhynchosporium* PKS lacking the MT domain (I: PKS8, IV: PKS10, PKS18). Most *Rhynchosporium* PKS were in reducing subclade II, which lacks the ER domain and contains all PKS-NRPS hybrid enzymes. Notably, *R. commune*-specific PKS13 appears to be closely related to LovB, one of the enzymes involved in the biosynthesis of lovastatin [84] in *Aspergillus terreus* (47.3% identity, 63.5% similarity, 6.7% gaps), suggesting that both enzymes catalyze similar reactions. Lovastatin is an inhibitor of 3-hydroxy-3-methylglutaryl-CoA reductase, which catalyzes a crucial step in the biosynthetic pathway leading to ergosterol (antifungal properties) or cholesterol (hypolipidemic agent; [85]). Two *Rhynchosporium* enzymes, PKS3 and PKS4, occur in the non-reducing clades II and III, respectively. Like all other *Rhynchosporium* PKS, however, the latter contain the DH reduction domain and therefore should be called partially reducing PKSs. Finally, PKS12 groups with *C. heterostrophus* PKS24 in the bacterial PKS clade, both being NRPS-PKS hybrids (*cf.* also NRPS below) and suspected of being horizontally transmitted from bacteria to fungi [86].

Among the key genes of secondary biosynthesis in *Rhynchosporium* those coding for NRPS and NRPS-like proteins represent the second largest group. These enzymes typically consist of one or more modules comprising an amino acid-adenylation domain (A), a thiolation domain (PP) and a condensation domain (C). Mono-modular NRPS4, tri-modular NRPS3 and tetra-modular NRPS2 were present in all species, whereas hexa-modular NRPS1 was missing in *R. orthosporum* (Table 6). Remarkably, *R. commune* isolate AU2 was again unique by carrying a specific gene coding for the most complex enzyme, the octa-modular NRPS5. Orthologs of the eight genes coding for NRPS-like (NPSL) proteins were found in all *Rhynchosporium* species with the exception of *NPLS4*, which was missing in *R. orthosporum*. In comparison to *Rhynchosporium*, closely related *M. brunnea* had orthologs of only six of the *NRPS* and *NPSL* genes. Only one *NRPS* gene shows isolate-specific occurrence indicating a possible role of its product in host specificity at the isolate level. In addition, several genes may be involved in BCG specificity.

**Table 6:**
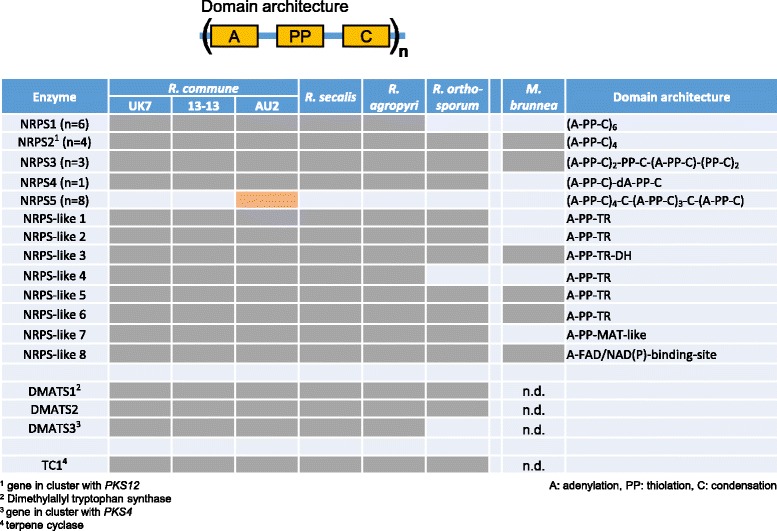
Nonribosomal peptide synthetases

**Fig. 8:**
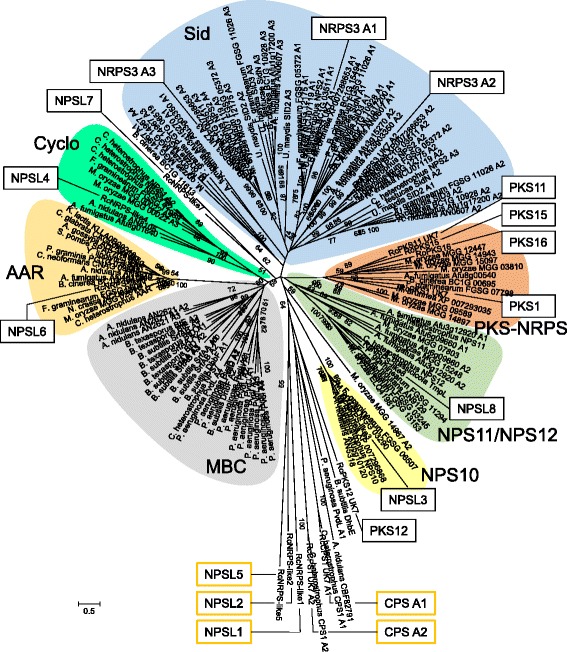
NRPS phylogenetic tree 1. The amino acid sequences of the A domain from 153 mostly mono-modular fungal and bacterial NRPS were used to construct the phylogenetic tree. Numerals at the nodes represent the percentages from 500 bootstraps. Numeral <50 were omitted. Scale: number of substitutions per nucleotide. Colored backgrounds indicate enzyme groups: Sid, siderophore synthetases; NPS11/NPS12, NPS11/12-like NRPS and ETP toxin synthetases; Cyclo, cyclosporine synthetases; MBC, major bacterial clade; AAR, α-amino-adipate reductases; NPS10, NRPS 10-like NRPS; PKS-NPS, hybrid enzymes. Orange-framed boxes mark members of outgroups

**Fig. 9:**
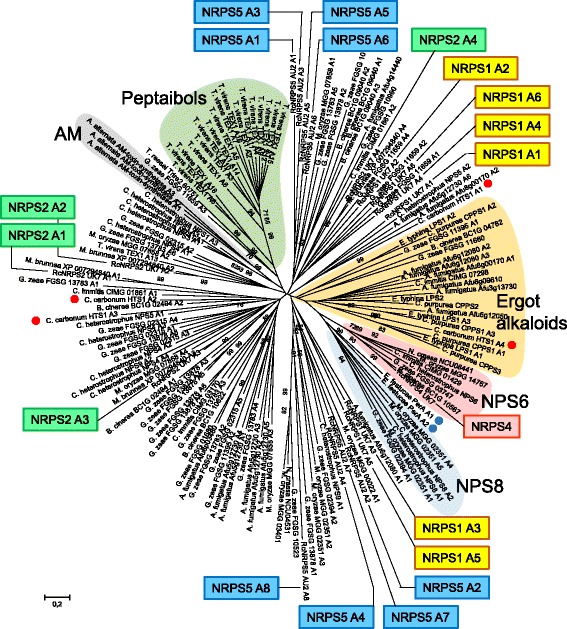
NRPS phylogenetic tree 2. The amino acid sequences of the A domain from 151 mostly oligo-modular specifically fungal NRPS were used to construct the phylogenetic tree. Numerals at the nodes represent the percentages from 500 bootstraps. Numerals <50 were omitted. Colored backgrounds indicate enzyme groups: *blue*, NPS8-like; *pink*, NPS6-like, *orange*, ergot alkaloid synthetases, *green*, peptaibol synthetase TEX1; *grey*, AM-toxin synthetase. *Red* and *blue* dots mark the modules of HC-toxin and peramine synthetase, respectively. Coloured boxes indicate modules of the 4 *Rhynchosporium* NRPS

Neither structures nor functions of the NRPS products in *Rhynchosporium* spp. are known. Therefore, the domain structures and, in particular, the A domains of the *Rhynchosporium* NRPS and NRPS-like proteins were compared to those of other fungi and bacteria. In a previous phylogenomic analysis of fungal NRPS and NRPS-like (NPSL) proteins [86] two main enzyme groups had been identified, one group containing seven subfamilies of primarily mono- and bi-modular fungal enzymes along with the bacterial clade (Fig. 8), the other group containing two subfamilies of primarily multi-modular and exclusively fungal (*Euascomycetes*, EAS; Fig. 9) enzymes. Members of the latter are presumed to be of more recent origin, most of them having less conserved structures suggesting the biosynthesis of products with more specific functions in niche adaptation including virulence. In contrast, members of the former group are presumably of more ancient origin, exhibiting more conserved domain architectures suggestive of more basic functions.

Following the previous analysis [86], the A domains of NRPS from *Rhynchosporium* were compared to those of other fungi including the closely related species *M. brunnea* and bacteria. Most NRPS subfamilies contain at least one member from the *Rhynchosporium* genus. Two proteins, tri-modular RcNRPS3 and mono-modular RcNRPS4, are predicted to be involved in the biosynthesis of two types of Fe^3+^-chelating hydroxamate-type siderophores required for iron uptake and storage. RcNRPS3 and its *M. brunnea* ortholog (XP_007288653) group with type-IV siderophore synthetases [87] (Fig. 8). Each of its three A domains joins a sub-branch with one of the A domains of *C. heterostrophus* NPS2, a ferrichrome synthetase. Ferrichromes, which are involved in intracellular iron storage, are typically cyclic hexapeptides built up of three *N* ^*5*^-acetyl-*N* ^*5*^-hydroxy-L-ornithines (AHO) and three small amino acids, an obligatory Gly and one to two Ser, Gly or Ala [87]. RcNRPS3 and its *M. brunnea* ortholog share the domain architecture with *C. heterostrophus* NPS2 including the C-terminal C-PP-C-PP module, which is predicted to function in ring closure of the nascent peptide [88]. In addition, the amino acid residues suggested to determine the substrate specificity of the A domains are almost identical in both enzymes and very similar to those of the A domains of *C. heterostrophus* NPS2 (Table 7). The NRPS2 product, ferricrocin, is an intracellular siderophore involved in iron storage, oxidative stress resistance, germination and sexual development in *A. nidulans* and other fungi [89]. This molecule is made up of two glycines and one serine in addition to the three AHO [90]. According to the 10-aa code for amino acid binding, the A1 domain of the NRPS from the three fungi binds glycine and the A2 domain binds serine [87, 91]. In the A3 domains of RcNRPS3 and its *M. brunnea* ortholog an L_239_ → M exchange has occurred compared to the A4 domain of *C. heterostrophus* NPS2. Nevertheless, the domain appears to be responsible for AHO binding (10-aa code: 9/10, 13-aa code: 11/13, 17-aa key positions: 14/17). Therefore, despite the presence of the second presumably glycine-binding domain A3 in *C. heterostrophus* NPS2, the siderophore product of RcNRPS3 and its *M. brunnea* ortholog is predicted to be identical with the NPS2 product, ferricrocin.

**Table 7:**
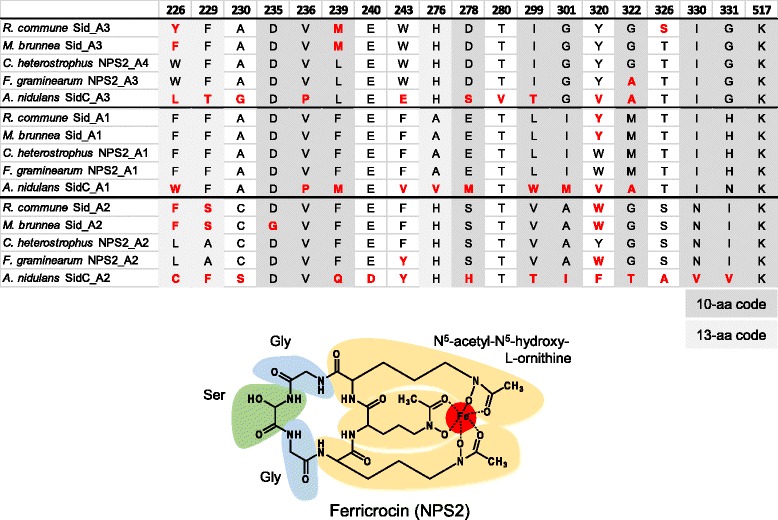
Amino acid code of siderophore synthases

RcNRPS4 was in the most conserved clade of the EAS group exemplified by *C. heterostrophus* NPS6 [86] coding for a different type of siderophore synthetases (Fig. 9). No *M. brunnea* homolog was identified. RcNRPS4 has the typical domain structure of this enzyme group, a single A-PP-C module followed by a module with a degenerate A domain (dA-PP-C). NPS6 of *C. heterostrophus* was shown to be responsible for the synthesis of extracellular siderophores of the coprogen family [92]. Deletion of the *NPS6* gene and its orthologs in different plant pathogenic fungi resulted in reduced virulence and hypersensitivity to H_2_O_2_.

Mono-modular RcNPSL3 and its *M. brunnea* ortholog belong to the NPS10 subfamily, which also has a conserved domain structure. The A-PP domain is followed by a thioester reductase (TR)-like domain and a dehydrogenase domain. RcNPSL4 has a single A-PP-TR module and groups together with mono-modular enzymes from *A. nidulans* (AN8105) and *A. fumigatus* (Afu8g01640) with the cyclosporin synthetases.

RcNPSL6 is in a clade with high bootstrap support that comprises α-aminoadipate reductases (AAR) such as Lys2 from *S. cerevisiae* and Lys1+ from *S. pombe*. AAR are evolutionary related to but not identical with NRPS and catalyze a step in the fungi-specific biosynthesis of lysine [93]. Neither RcNPSL7 nor *B. cinerea* protein BC1G_11613 fits into one of the clades described. Both proteins have the same domain architecture. The A and PP domains are followed by a domain that makes up the C-terminal half of the protein, is predicted to contain three pairs of transmembrane domains (TMDs) and is exclusively found in NRPS.

RcNPSL8 is in the same clade as *C. heterostrophus* NPS12 and *Alternaria brassicicola* TmpL (with high bootstrap support). These proteins lack thiolation (PP) and condensation (C) domains and, hence, are no true NRPS proteins. Instead, they are characterized by an A domain and a C-terminal FAD/NAD(P)-binding domain, which are separated by putative TMDs. The number of TMDs is predicted to be between 5 and 7 (depending on the exclusion or inclusion of two TMDs with low probability following TMD1). Therefore, membrane orientation of these proteins needs to be verified experimentally. TmpL was shown to be involved in virulence through regulation of intracellular ROS concentrations and tolerance to external ROS [94]. The biochemical function of the TmpL-like proteins is not known, but they are suggested to possess an enzymatic function involving FAD/NAD(P) binding like other flavoenzymes such as enzymes from the ferric reductase group.

The basic architecture of NPSL1-6 is very similar with A- and PP-domains followed by a TR domain. However, the A domains of RcNPSL3, RcNPSL4 and RcNPL6 are structurally similar to the A domains of RcNRPS1-5 (NCBI PSSM-ID CD05930), but deviate from those of RcNPSL1 (CD04433), RcNPSL2 (CD05907) and RcNPSL5 (CD05936). As a consequence, the latter along with RcPKS12 (NRPS-PKS hybrid, CD04433) form a phylogeny outgroup. Other outgroup A domains comprise the *Rhynchosporium* ortholog of CPS1, a protein consisting of two A domains (A1: CD05931, A2: CD05905), which was shown to be a virulence factor of *C. heterostrophus* and *Gibberella zeae* on maize and of *C. victoriae* on oats [95]. Furthermore, A domains from bacterial proteins, *B. subtilis* 2,3-dihydroxybenzoate-AMP ligase (DhbE, CD5920) and *P. aeruginosa* pyoverdine synthetase (PvdL A1, CD05931), as well as an *A. nidulans* NPSL protein, CBF82791 (CD05906), were used as outgroup.

In the mostly multi-modular EAS group (Fig. 9), little information can be deduced from the phylogenetic position of the A domains regarding the function of NRPS products. However, occurrence of the A domains of tri-modular AM-toxin synthetase from *A. alternata* or 14 of the 19 modules of peptaibol synthetase Tex1 from *Trichoderma virens* in a single subclade with high bootstrap support is suggestive of an evolutionary origin of the enzymes by duplication events. A similar mechanism may have played a role in the evolution of *Rhynchosporium* NRPS enzymes. Three of the A domains of hexa-modular RcNRPS1 occur in one subclade, two in another subclade and one in a third subclade. Similarly, the A domains of octa-modular RcNRPS5 are in three subclades with four, three and one members, respectively. In contrast, the A domains of tetra-modular RcNRPS2 and its *M. brunnea* ortholog are spread over four different subclades indicating a recombination-type mode of molecular evolution.

Finally, the four PKS-NRPS (PKS1, PKS11, PKS15, PKS16) from *Rhynchosporium* and the PKS1 ortholog from *M. brunnea*, which all belong to (partially) reducing PKS clade II (*cf.* PKS above), are found in the PKS-NRPS subfamily of the phylogenetic tree (Fig. 8). They all have the same domain architecture except for RcPKS16, which lacks the C-terminal PP domain (for review s. [96, 97]).

**Fig. 10:**
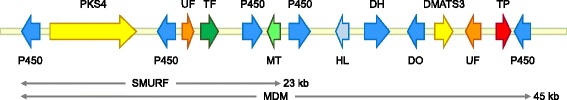
*PKS4* gene cluster of *R. commune*. The cluster harbors genes coding for two secondary metabolism key enzymes, PKS4 and DMATS3, along with several decorating enzymes, a putative transporter and a regulatory gene. DH, dehydrogenase; DO, dioxygenase; HL, hydrolase; MT, methyl transferase; P450, cytochrome P-450 enzymes; TF, transcription factor; TP, transporter; UF, unknown function. SMURF and MDM, see text

The basic structures formed by the key secondary biosynthesis enzymes are usually modified by decorating enzymes such as oxidoreductases, glycosyl or prenyl transferases. Their genes are frequently found in contiguous clusters, i.e., most or all genes of a biosynthetic pathway, possibly along with genes responsible for regulation and transport, are co-localized and co-regulated [98]. For instance, the cluster for the biosynthetic pathway of the polyketide asperfuranone in *A. nidulans* comprises genes coding for two fungal PKSs, five additional enzymes and a transcriptional activator [99]. Similarly, putative *PKS* gene clusters were detected in the *Rhynchosporium* BCG genomes (Additional file 7: Table S5). Two clusters contain genes coding for the key biosynthetic enzymes of two different secondary metabolite classes: the *PKS4* cluster includes the *DMATS3* gene (Fig. 10) and the *NRPS2* cluster harbors the *PKS12* gene. DMATS3 might be a transferase required for the prenylation of a PKS4-derived polyketide. Alternatively, the gene pairs might be involved in the biosynthesis of mixed compounds through separate assembly lines.

### Small secreted proteins: necrosis-inducing proteins

Secreted effector molecules have been recognized as crucial for host colonization and pathogenicity [100]. In both pathogens and mutualists they function by deregulating the host immune response. In addition, they have a role in self-defense and competition with other microbes [101, 102]. Furthermore, they influence the physiology of the host in favor of the pathogen’s needs, thus enhancing virulence [103]. Finally, they contribute to host adaptation and to establishing host specificity, thereby driving microbial speciation [104].

**Fig. 11:**
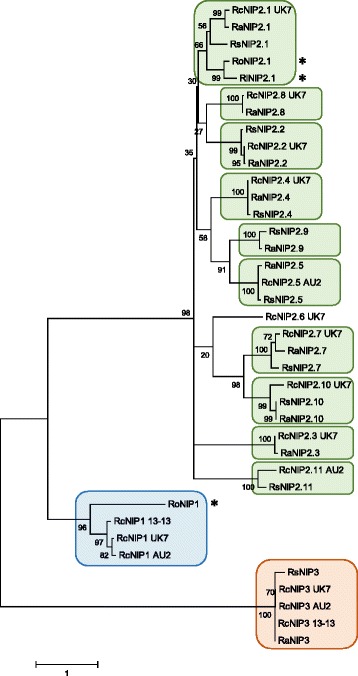
Phylogeny of NIPs. The amino acid sequences of 39 mature NIPs were aligned using the MUSCLE algorithm. For clarity reasons and due to their high similarity only one NIP2 protein from the 3 *R. commune* isolates was taken into consideration. The tree is drawn to scale. Numerals on the nodes represent the percentages from 500 bootstraps. Asterisks mark the CCG species. Scale: number of substitutions per nucleotide

In *R. commune*, the genes *NIP1*, *NIP2* and *NIP3* code for effector proteins that contribute quantitatively to the virulence of *R. commune* depending on the host cultivar [105]. Therefore, the sequenced genomes of all *Rhynchosporium* species were screened for the presence of orthologous genes using the *NIP* gene sequences from *R. commune* isolate UK7 as probes. In this context, only those *R. lolii* genes that have homologs in *R. orthosporum* could be included. *NIP1* and *NIP3* occur as single genes in the genomes. *NIP3* orthologs coding for highly similar proteins are present in the three BCG species, whereas *NIP1* orthologs are found in *R. commune*, albeit not in all isolates (*cf.* [106]), and in the CCG species *R. orthosporum* (Fig. 11). *NIP2* is also a single gene in the two CCG species. In contrast, *NIP2* families of 7-10 members are present in the BCG species (*NIP2.1*-*NIP2.11*).

**Fig. 12:**
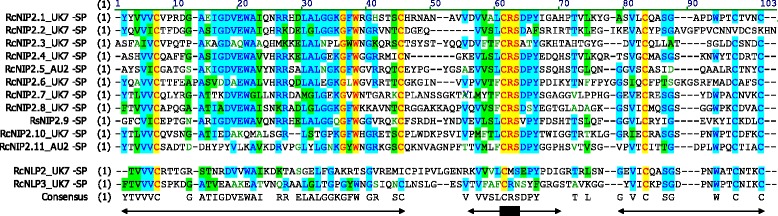
Sequence comparison of NIP2 and NIP2-like proteins (NLP). For clarity reasons the amino acid sequences of the mature proteins (-SP) from *R. commune* UK7 were aligned with the sequences only available in other isolates/species. Arrows indicate the domains identified by MEME. The CRS domain in position 61-63 (marked by the black box on the arrow) differentiates the two protein groups

Comparison of the deduced NIP2 amino acid sequences using Multiple Expectation Maximization for Motif Elicitation (MEME, version 4.9.1; [107]) revealed three conserved protein motifs, a 40-amino acid stretch spanning the N-terminal half of the protein, followed by a 15-amino acid domain around an absolutely conserved CRS motif, and near the C-terminus of about 50% of the proteins another 15-amino acid sequence. In addition, BLASTp analysis identified genes coding for proteins with similarity to NIP2 in the BCG genomes. These NIP2-like proteins (NLPs) differ from NIP2 by a smaller degree of domain conservation, but most strikingly by the absence of the CRS motif, which is absolutely conserved in NIP2 (Fig. 12). All NIP2 and NLP carry six cysteine residues at homologous positions. Interestingly, all proteins except NIP2.2 have a cysteine at the C-terminus. This structural feature was also found in NIP1. The phylogenetic tree of the mature NIP2 proteins demonstrates that the different isoforms are more similar between fungal species than they are to each other. This implies that duplication of the *NIP2* gene occurred in a common ancestor before the three BCG species diverged, possibly to allow or facilitate host jump and colonization of new cereal crop species.

To date no biochemical function could be assigned to NIP2. In contrast, NIP1 and NIP3 both stimulate the host plant plasma membrane H^+^-ATPase [108]. This enzyme generates a cellular membrane potential, which may affect nutrient accumulation and fungal membrane transport processes. In addition, acidification of the apoplast may create more convenient growth conditions for the fungus [109]. The originally reported induction of necrosis by the purified proteins [110] may therefore be a consequence of over-stimulation of the H^+^-ATPase due to severe overloading of the host leaf tissue [23]. An amino acid sequence comparison of NIP1 and NIP3 revealed that a stretch of six amino acids, -G_45_EGGNG_50_-, which is present in exposed loop III near the C-terminus of RcNIP1 [111], but absent from RoNIP1 (50.8% similarity, 39.7% identity with RcNIP1), is almost identically found near the C-terminus of NIP3 as -G_82_EGGEG_87_-. Remarkably, G_45_ is essential for NIP1 activity [7, 112], which suggests that this amino acid motif could be critical for the common function of NIP1 and NIP3.

NIP1 from *R. commune* is targeted by the barley resistance gene *Rrs1* [7], thereby triggering defense reactions in the host [113]. To be able to infect *Rrs1*-barley the fungus therefore evolved two strategies to prevent recognition of NIP1, namely introduction of mutations into the *NIP1* gene and its complete deletion [106, 112]. As the consequence, *NIP1* was only found in about half of the *R. commune* isolates analyzed and inactive NIP1 isoforms were identified [7, 112]. Nevertheless, the importance of NIP1 as a virulence factor of *R. commune* was shown in a recent study on the quantitative virulence of 14 fungal isolates from each of nine global field populations worldwide. As the result, “on average isolates carrying a functional NIP1 protein had significantly higher virulence than isolates where the protein was missing or non-functional” [114]. In contrast to *NIP1*, *NIP3* was found in almost all *R. commune* isolates studied [106] as well as in *R. secalis* and *R. agropyri*. Hence, at least one of the H^+^-ATPase stimulators, NIP1 or NIP3, appears to be present in a given fungal species/isolate.

To give a résumé, *NIP1* and *NIP3* appear to be specific for *R. commune* and the BCG species, respectively. The presence of an – albeit possibly “inactive” – NIP1 in *R. orthosporum* indicates that the gene may have been lost in the other species. In contrast, each of the five *Rhynchosporium* species harbors at least one *NIP2* gene. However, evolution of the *NIP2* gene family by gene duplication and sequence diversification [115] suggests an involvement of this protein in the adaptation of the BCG species to their hosts.

### Small secreted proteins: specific occurrence of effector candidates in *R. commune*

The NIP effectors had been identified originally through their necrosis-inducing activity [110]. With the genome sequences available, additional candidate effector genes were obtained through an *in silico* approach. To this end, several general criteria based on existing knowledge about protein effectors from other fungal pathogens were established that ought to be fulfilled by candidate effector proteins (*cf.* [116, 117]). Effectors are secreted, i.e. most of them carry a signal peptide while lacking an additional transmembrane domain. Furthermore, they are usually of small size and cysteine-rich. In addition, they should be specific for the genus *Rhynchosporium* and expressed during pathogenesis, probably during the stage of fungal establishment *in planta*. Of particular interest are those effector genes that are present exclusively in the genome of a single species and, hence, may be involved in determining host specificity of this fungal species.

**Fig. 13:**
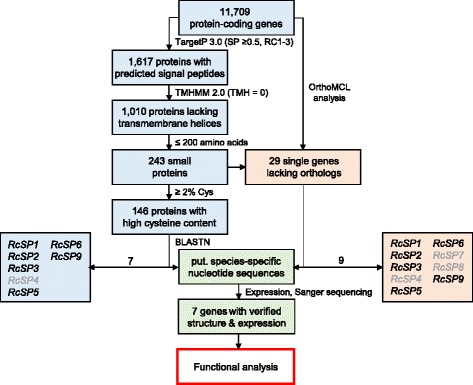
Flow chart for the identification of candidate effector genes in the *R. commune* genome (for details see text). RcSP7 and RcSP8 did not match the ≥ 2% Cys criterion. *RcSP4* (mis-annotated), *RcSP7* (very low expression) and *RcSP8* (no expression) were not submitted to functional analysis

The genome of *R. commune*, isolate UK7, was screened for candidate genes coding for proteins that match the general criteria (Fig. 13). The subgroup containing ≥2% cysteine residues contained 139 proteins. The occurrence of these genes was probed (BLASTn) in the genomes of the other *Rhynchosporium* species (with the exception of *R. lolii*) to identify candidates, which may be crucial for host specificity. In *R. commune*, seven gene models (*RcSP1, RcSP2, RcSP3, RcSP4, RcSP5, RcSP6, RcSP9*) were found to be specifically present, among them the *NIP2.6* paralog (*RcSP6*). When the OrthoMCL algorithm [118] was applied to the *Rhynchosporium* genomes, 29 *R. commune* proteins were found to lack orthologs in the other species. BLASTn analysis identified the seven *RcSP* genes and two additional genes in the <2%-cysteine fraction (*RcSP7*, *RcSP8*). No candidate proteins were identified in the 201–350 amino acid size fraction.

The gene models as predicted by the annotation algorithms were verified using EST libraries from *R. commune* isolate UK7 grown in liquid culture (A. Navarro-Quezada and W. Knogge, unpublished), from germinated conidia of British *R. commune* isolate 214 and from epidermal strips of barley leaves 3 dpi with British *R. commune* isolate 2LA (A. Avrova, unpublished). In addition, RT-PCR was carried out using gene-specific primers and RNA from *ex planta* grown *R. commune* isolate UK7 as a template. Sanger sequencing of the amplification products confirmed the structures of seven of the *R. commune* genes (*RcSP1*, *RcSP2*, *RcSP3*, *RcSP5*, *RcSP6, RcSP7, RcSP9*). In contrast, *RcSP4* had been annotated incorrectly, while *RcSP8* was not expressed. More detailed BLASTn and OrthoMCL analysis detected paralogs of *RcSP1*, *RcSP2*, *RcSP3* and *RcSP5* in *R. commune* and homologs in other *Rhynchosporium* species (Table 8, Additional file 8: Table S6). The original genes were renamed accordingly.

**Table 8:** *RcSP* homologs

*R. commune* protein	Identity [%]	Orthologs		
		*R. secalis*	*R. agropyri*	*R.orthosporum*
RcSP1.1	100			
RcSP1.2	50.0			
RcSP1.3	27.6	RsSP1.3 (44.9)		
RcSP2.1	100			
RcSP2.2	44.4		RaSP2.2 (82.1)	
RcSP3.1	100		RaSP3.1 (63.4)	RoSP3.1 (47.5)
RcSP3.2	46.5	RsSP3.2 (45.5)	RaSP3.2 (46.6)	RoSP3.2a (31.3) RoSP3.2b (42.4)
RcSP3.3	57.4		RaSP3.3 (98.1)	RoSP3.3 (47.0)
RcSP5.1	100			
RcSP5.2	28.0		RaSP5.2a (98.2) RaSP5.2b (51.0) RaSP5.2c (55.8)	
RcSP5.3	25.3		RaSP5.3 (52.5)	RoSP5.3 (37.2)
RcSP5.4	26.7	RsSP5.4 (78.9)	RaSP5.4 (75.0)	

Numbers in brackets indicate the amino acid identity (%) with the respective *R. commune* protein

**Fig. 14:**
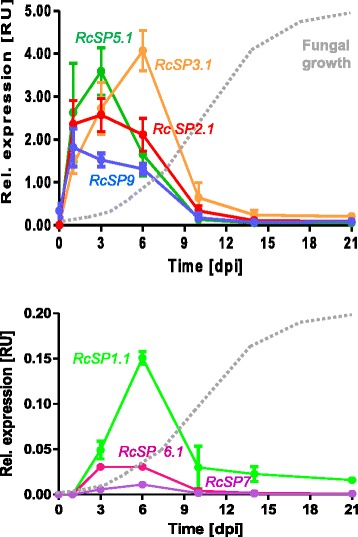
Expression of *RcSP* genes during pathogenesis. Relative RNA abundance was measured by qRT-PCR during growth of fungal isolate UK7 on barley cv. ‚Ingrid’. Dotted line indicates the development of fungal biomass

Expression of the seven *R. commune* candidate effector genes during pathogenesis was quantified using RNA extracted from inoculated leaves of susceptible barley cultivar ‘Ingrid’ at different time-points post inoculation with *R. commune* isolate UK7 [105]. Transcript amounts of the target genes were determined relative to the transcript amounts of a constitutively expressed fungal gene, *GPD*, by qRT-PCR [119]. As observed for the *NIP* effector genes [105], the *RcSP* genes were all transiently expressed early during fungal infection of susceptible host leaves. Between 10 and 14 dpi, when disease symptom occur, *RcSP* mRNAs virtually can no longer be detected (Fig. 14). *RcSP2.1, RcSP5* and *RcSP9* showed a maximum transcript abundance at 3 dpi, whereas *RcSP1.1* and *RcSP3.1* mRNA levels reached a maximum at 6 dpi. However, *RcSP1.1* mRNA abundance accounted for only about 4% of that of *RcSP3.1. RcSP6* and *RcSP7* mRNA levels were even lower, peaking at 3–6 dpi. When the infection process of the hemibiotroph *Colletotrichum higgensianum* on *Arabidopsis thaliana* was studied successive waves of effector gene expression could be observed during the consecutive developmental stages from unpenetrated spores through penetrated biotrophic hyphae to the biotrophy to necrotrophy switch [120]. The different stages of *Rhynchosporium* development cannot be discerned as clearly as in *C. higgensianum*. Nevertheless, maximum transcript levels for some genes (*RcSP2.1*, *RcSP5*, *RcSP9, NIP1*) occur at 1-3 dpi, for others (RcSP1.1, RcSP3.1, NIP2, NIP3) at 6 dpi suggesting a similar sequence of gene expression waves.

**Fig. 15:**
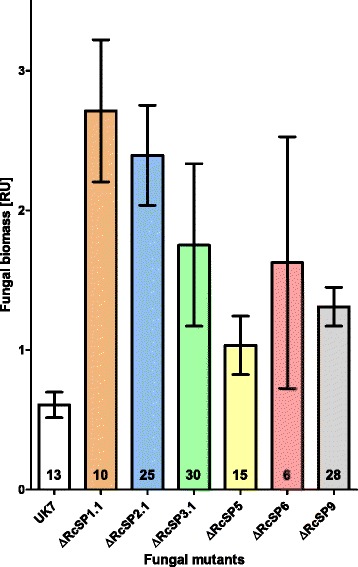
Growth acceleration of deletion mutants. Relative biomass of fungal deletion mutants and wild-type isolate UK7 was determined by qPCR at 14 dpi on barley cv. ‘Ingrid’. Results from independent mutants are combined (cf. Additional file 9: Figure S3). Bars represent the 95% confidence intervals. *n*-values are given at the base of the bars

**Fig. 16:**
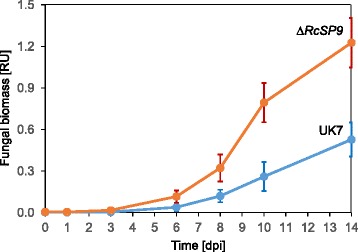
Growth of Δ*RcSP9* mutants. Relative biomasses of three independent deletion mutants and wild-type isolate UK7 were determined by qPCR during pathogenesis on barley cv. ‘Ingrid’. Bars represent 95% confidence intervals. n_Δ*RcSP9*_ = 9, n_UK7_ = 4

**Fig. 17:**
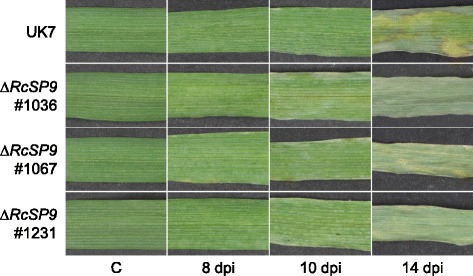
Disease phenotype of RcSP9 deletion mutants. Primary leaves of barley cv. ‘Ingrid’ were inoculated with spores of wild-type isolate UK7 or of the mutants (#1036, #1067, #1231) and photos were taken at indicated times post inoculation. C, mock inoculation

To assess the role of the *R. commune*-specific effector genes during pathogenesis, deletion mutants were generated. Using split-marker recombination [121] single deletion mutants were obtained for *RcSP1.1* and *RcSP6*, two independent mutants for *RcSP5.1* and three for *RcSP2.*1, *RcSP3.1* and *RcSP9*. After inoculation of susceptible barley cultivar ‘Ingrid’ none of the deletion mutants caused a phenotype deviating substantially from that caused by wild type isolate UK7 at 14 dpi (not shown). However, when fungal biomass *in planta* was quantified by qPCR 10 of the 13 individual *RcSP* deletion mutants had grown to a significantly greater amount (1.7–4.5-fold) than the wild type UK7 (Fig. 15, Additional file 9: Figure S3), whereas deviations from wild type did not occur during fungal growth on agar plates (not shown). Their overlapping 95% confidence intervals allowed to combine the data of the independent deletion mutants of single *RcSP* genes (Fig. 15). The weakest growth response occurred with the Δ*RcSP5* mutant, whereas the largest confidence interval was found with the Δ*RcSP6* mutant. The latter finding may be due to the fact that *RcSP6* is identical with *NIP2.6*, which is part of a gene family with seven additional members in *R. commune* isolate UK7. The observed growth increase of the deletion mutants is in contrast to the *NIP* gene deletion mutants, which did not differ from wild type (Δ*NIP1*, Δ*NIP2*) or grew substantially slower (Δ*NIP3*) than the wild type on highly susceptible cultivar ‘Ingrid’. (All three mutants showed strong growth reduction on moderately susceptible barley cultivar ‘Atlas’; [105]). When growth of the three *RcSP9* deletion mutants and that of wild type were compared in more detail during pathogenesis, mutant biomasses deviated significantly from that of isolate UK7 from about 6 dpi on (Fig. 16, Additional file 10: Figure S4). In addition, close inspection of disease phenotype development revealed that the first symptoms became visible already at 8–10 dpi with the deletion mutants, whereas wild type symptoms did not occur before 10 dpi (Fig. 17; Additional file 11: Figure S5). At 14 dpi the mutant phenotype appeared more severe than the wild type phenotype; almost the entire upper blade of the mutant-inoculated leaves was necrotic, whereas green tissue remained between the lesions of wild type-infected leaves.

In summary, significant quantitative effects on fungal growth and symptom development became apparent upon deletion of the six *R. commune*-specific genes. Notably, the deletion-caused growth acceleration suggested that the fungus is not adapted for the most rapid development *in planta*. This is similar to mutants of the grass endophyte *Epichloë festucae*, which had switched from mutualistic to pathogenic growth including a dramatically increased fungal biomass on perennial ryegrass. Different types of non-effector proteins were inactivated in the mutants; a MAP kinase (SakA, [122]), an NADPH oxidase (NoxA, [123]), which is regulated by NoxR [124] through interaction with small GTP binding protein RacA [125], and a siderophore-synthesizing NRPS (SidN, [126]). Therefore, it was postulated that functional stress signaling, regulated ROS synthesis and siderophore-mediated iron homeostasis are crucial for restricting fungal growth as prerequisite for maintaining the mutualistic interaction with the host. It is tempting to speculate that the *R. commune* RcSPs fulfil an analogous role in affecting hyphal growth, probably by interacting with plant compounds, thereby establishing an endophyte-like growth in host leaves during the early symptomless period of pathogenesis.

It remains to be shown whether the effects caused by the RcSPs contribute to host specialization. However, the occurrence of specific effector genes in the genome of one *Rhynchosporium* species and their absence from others species represents the simplest model to explain host specificity. Alternatively, effector genes, albeit present in several *Rhynchosporium* genomes, may be specifically expressed only in the interaction of one of them with its host plant and not in the specific interactions of other species. Finally, host specificity may originate from structural differences of effector proteins rather than from presence or expression of specific genes. In addition, small molecules such as host-specific toxins originating from fungal secondary metabolism may play a role in colonizing a particular host species.

## Conclusions

Phylogenetic analysis confirmed that the *Rhynchosporium* genus belongs to the *Leotiomycetes* clade of fungal systematics, most likely to the *Dermataceae* family, the BCG and CCG species clearly forming separate branches of the *Rhynchosporium* genus. Genome-wide SNP analysis along with the structure of the rDNA regions suggested that during BCG evolution *R. agropyri* branched off shortly before *R. commune* and *R. secalis* diverged. At least in the BCG species, complete mating type loci exist. In addition, core meiotic genes are present and expressed along with most genes required for meiosis and sexual recombination in other (ascomycete) fungi, providing molecular evidence for the existence of a cryptic sexual cycle.

Bioinformatic analysis revealed a large number and types of CWDEs, which are characteristic for hemibiotrophic and necrotrophic fungi infecting monocotyledonous hosts, but failed to provide evidence for their involvement in host specialization. In contrast, the occurrence of genus-, species- and isolate-specific *PKS* and *NRPS* genes suggests a possible role in host adaptation in particular of those genes, which are missing from the genome of the closest sequenced relative, the poplar pathogen *M. brunnea*. The most important finding was, however, that six *R. commune*-specific effector proteins contribute to decelerating fungal development *in planta*. Host adaptation of this hemibiotrophic fungus therefore appears to aim at stabilizing the biotrophic growth stage in favor of the necrotrophic destructive stage. Unlike haustoria-forming fungi, apoplastic fungi like *R. commune* colonize the intercellular matrix of their hosts relatively slowly, usually without triggering a fast hypersensitive cell death response. The long biotrophic stage of *Rhynchosporium* development resembles endophytic fungal growth, rendering this fungus a possible object for studying the mutualism-parasitism transition.

## Methods

### Fungal strains

The following fungal isolates were subjected to genome sequencing; for *R. commune* UK isolates UK7 and 13-13 and Australian isolate AU2 [21], for *R. secalis* Swiss isolate 02CH4-6a.1 [17], for *R. agropyri* Swiss isolate 04CH-RAC-A.6.1 (B. McDonald, ETH Zurich), for *R. lolii* UK isolate 15lp11 [16], and for *R. orthosporum* Swiss isolate 04CH-BAR-A.1.1.3 (B. McDonald, ETH Zurich).

### Fungal cultures, DNA extraction, genome sequencing and assembly

Fungal culture conditions, DNA extraction and genome sequencing were described previously [30]. Briefly, the genomes of *R. commune*, isolates UK7 and 13-13 and *R. secalis* were sequenced using the Roche 454 Genome Sequencer FLX with GS FLX Titanium series reagents, fosmids and the lllumina Genome Analyzer IIx (paired-end and mate-pair libraries). Paired-end libraries of *R. commune*, isolate AU2, *R. agropyri* (along with a mate-pair library) and *R. orthosporum* were sequenced using Illumina GAIIx and HiSeq. To establish the genome of *R. commune*, isolate UK7, as the reference genome, contigs were assembled using the ALLPATHS-LG software package [127] and pre-assembled contigs were combined into scaffolds using the SSPACE algorithm [128]. The same strategy was followed to assemble the genome of *R. agropyri*, whereas genome assembly for *R. commune*, isolate AU2 and for *R. orthosporum* was carried out using the Velvet algorithm package [129], and for *R. secalis* using the Newbler software package. For *R. commune* isolate 13-13, 454 and Illumina GAIIx reads were assembled independently using the Newbler and Velvet algorithm packages, respectively, followed by combined assembly using Minimus2 [130]. The genome of isolate 15lp11 from the fifth species, *R. lolii* [16], was sequenced by Richard Harrison (Illumina MiSeq, East Malling Research, East Malling, UK) and a draft assembly using the VELVET algorithms [129] was obtained by David Hughes (University of Hertfordshire, Hatfield, UK). Sequence reads of this isolate were mapped to the *R. orthosporum* genomic contigs. Hence, differential sequence information could be identified only for *R. lolii* genes that are orthologous to *R. orthosporum* genes.

### Structural annotation

Gene models for *R. commune* UK7, *R. secalis* and *R. agopyri* were generated by three *de novo* prediction programs: 1) Fgenesh [131] with different matrices (trained on *Aspergillus nidulans*, *Neurospora crassa* and a mixed matrix based on different species), 2) GeneMark-ES [132] and 3) Augustus [133] with *R. commune* ESTs as training sets. Annotation was aided by exonerate [134] hits of protein sequences from *B. cinerea* T4, *B. cinerea* B05.10, *S. sclerotinia, R. commune* UK7, *R. secalis* and *R. agropyri*, respectively, to uncover gene annotation gaps. EST transcripts were assembled and mapped on the genome using Blat, a final cleaning was done with Perl Scripts. The different gene structures and evidences (exonerate mapping and EST transcripts) were visualized in GBrowse [135], allowing manual validation of coding sequences. The best fitting model per locus was selected manually and gene structures were adjusted by splitting or fusion of gene models or redefining exon-intron boundaries if necessary. The final call sets comprises 12,212 (*R. commune* UK7), 13,151 (*R. secalis*) and 13,674 (*R. agropyri*) protein coding genes. In addition, 91 (+22 mitochondrial) tRNA-encoding genes are predicted for *R. commune* UK7, 95 (+22 mitochondrial) for *R. secalis* and 107 for *R. agropyri* using tRNAscan-SE [136]. The predicted protein sets were searched for highly conserved single (low) copy genes to assess the completeness of the genomic sequences and gene predictions. Orthologous genes to all 246 single copy genes were identified for all three proteomes by Blastp comparisons (eVal: 10^−3^) against the single-copy families from all 21 species available from the FUNYBASE [137]. In addition, all of the 248 core genes commonly present in higher eukaryotes (CEGs) could be identified by Blastp comparisons (eVal: 10^−3^) for two proteomes, whereas only one protein was missing in the *R. agropyri* proteome [138].

### Identification of repetitive DNA elements

Determination of repeat sequences involved first the calculation of repeat families *de novo* followed by assessment of known repeat elements in a second step. Families of previously unknown interspersed repeat elements were identified by RepeatScout [139]. Repeat families were included when they comprised more than 10 repeats and when their consensus sequence length was longer than 50 bp. In addition, low complexity and simple sequence repeats were determined with the tools NSEG [140] and Tandem Repeats Finder [141], which are part of the RepeatScout procedure and remove them from the interspersed repeat library.

The RepBase database [142] was used to detect previously published families of transposable elements, pseudogenes and retroviruses. In order to determine the exact locations of the repetitive elements on the genome we used the RepBase library and the calculated library of interspersed repeat families as input for RepeatMasker [143]. RepeatMasker was also used to find and mask genomic regions of low complexity. We applied the automated classification tool TEclass [144] to categorize the predicted repeat sequences into the four main transposable element categories DNA transposon, long interspersed nuclear element (LINE), short interspersed nuclear element (SINE) and retrotransposon with long terminal repeats (LTRs).

### Functional annotation of predicted open reading frames and data repositories

The protein coding genes were analyzed and functionally annotated using the PEDANT system [145]. Data sets are accessible at http://pedant.helmholtz-muenchen.de/genomes.jsp?category=fungal. The genome and annotation data were submitted to the European Nucleotide Archive (ENA, *R. commune*, isolate UK7: http://www.ebi.ac.uk/ena/data/view/FJUW01000001-FJUW01000164; *R. secalis*: http://www.ebi.ac.uk/ena/data/view/FJVC01000001-FJVC01002130; *R. agropyri*: http://www.ebi.ac.uk/ena/data/view/FJUX01000001-FJUX01000508).

### Phylogenetic analyses

Sequence data for phylogenetic estimations of fungal relationships were generated using standard PCR protocols and primers that were (a) designed according to the 18S rDNA sequence from *R. commune*, isolate 788 (GenBank accession no. AY038583.1, [34]; Additional file 12: Table S7) or (b) described in a previous study [35]. After amplification and cloning into pJET (Thermo Scientific, Schwerte, Germany) Sanger sequencing (LGC Genomics, Berlin) was done on the nearly full-length rDNA region (18S rDNA-ITS1-5.8 S rDNA-ITS2-28S rDNA) using five (*R. commune*, *R. secalis*), six (*R. agropyri*) or seven (*R. orthosporum*) overlapping primer pair combinations starting on the 5′ side with primer 18SrDNA1f and ending on the 3′ side with primer LR7 (Additional file 12: Table S7). The sequences coding for elongation factor 1-α (EF1-α) and the RNA polymerase II subunit RPB1 and RPB2 were extracted from the genome database.

To integrate the *Rhynchosporium* genus into the estimated fungal phylogeny [35], sequences coding for 18S rRNA, 28S rRNA, the ITS region including the 5.8S rRNA, *elongation factor 1-α* (*EF1-α*), *RPB1* and *RPB2* were obtained from GenBank (genome projects, AFTOL data) for 12 taxa of the *Leotiomycetes* class (Additional file 13: Table S8). Sequences for each gene were aligned using the ClustalW algorithm implemented in BioEdit (v7.2.5) and ambiguous regions were excluded. Gap-bridging sequences in the alignments were removed. Accordingly, an alignment utilizing the implemented ClustalW algorithm of MEGA (v6.06; [146] was generated, where sequences differ in single nucleotide polymorphisms (SNP) and in additional sequences of up to three consecutive nucleotides per site. The conflict that the *RPB1* sequences of the species *Coccomyces dentatus*, *Potebniamyces pyri*, *Monilinia fructicola* and *Lachnum virgineum* are not accessible was ignored. Data were combined in one matrix with concatenated trimmed sequences in the order 18S rDNA-28S rDNA-ITS-*EF1a*-*RPB1*-*RPB2*. For phylogenetic estimation the nucleotide sequences were imported into MEGA (v6.06) and fitted to a general-time-reversible model with a proportion of Invariant sites and Gamma distribution rates (four rate classes, GTR + I + Γ). The Maximum Likelihood method was applied using 1000 bootstrap replicates (MLBS). Gaps and missing data were treated as complete deletion. Nearest-Neighbor-Interchange (NNI) served as the Maximum Likelihood heuristic method. The MEGA-estimated phylogeny was verified with an independent MLBS estimation in PHYML [147], also under the GTR model with gamma distribution in four rate classes. 250 bootstraps were done and tree improvement used NNI and subtree pruning and regrafting (SPR).

In a second phylogenetic estimation, a tree was generated that includes more distantly related fungal species. To this end, the amino acid sequences of EF1-α, RBP1 and RBP2 from *R. commune* were submitted to BLASTp searches [148] to identify the homologous genes in 17 different taxa (Additional file 13: Table S8). An RBP1 sequence from *B. fuckeliana* was not found. The combined and concatenated sequence data were fitted into a single EF1-α/RPB1/RPB2 matrix. No trimming was performed due to the availability of complete coding sequence for all proteins investigated. Amino acid sequences were aligned using the ClustalW algorithm in MEGA (v6.06). Thus, phylogenetic estimation was carried out under the LG substitution model [149] utilizing gamma distributed rates with invariant sites (G + I, four discrete gamma categories). Maximum Likelihood analyses took 500 bootstrap replications of heuristic searches into account (MLBS). Partial deletion was estimated for gaps and missing data and tree improvement were accomplished using NNI. The MEGA-estimated phylogeny was verified by MLBS estimation in PHYML using the LG method with four substitution rate classes. 250 bootstraps were performed and tree improvement used NNI and SPR.

Genome-wide single nucleotide polymorphisms (SNPs) were used to infer the phylogeny of the closely related BCG species. To this end, the genomes of *R. commune*, isolates UK7, AU2 and 13-13, *R. secalis*, *R. agropyri* and *R. orthosporum* were aligned using the MAUVE algorithm (version 2.4.0; Darling lab, University of Technology, Sydney). The obtained collinear genomic sequence (24.85 Mb) represented 45–50% of the genomes. Homologous sites containing SNPs at least in one of the genomes were identified and concatenated. A multiple sequence alignment was generated and a phylogenetic tree was constructed under the Tamura-Nei substitution model ([150], G + I, four discrete categories). Maximum Likelihood analysis was done with NNI and 500 bootstrap replications. Gaps and missing data were eliminated. Divergence times were calculated for all branching points using the RelTime method [151]. Relative times were translated into absolute times based on previous Bayesian calculations [17].

To construct the phylogenetic tree of the NIPs the amino acid sequences of the mature proteins were used. For the phylogenetic trees of polyketide synthases and non-ribosomal peptide synthases, amino acid sequences from other mostly fungal taxa were obtained from the NCBI databases. Enzymatic domains were identified by BLASTp analysis using NCBI’s Conserved Domain Database [82] and aligned with MUSCLE [152]. Evolutionary analyses were done in MEGA (v6.06). The evolutionary history was inferred by using the Maximum Likelihood method based on the LG substitution model [149]. In each case the tree with the highest log likelihood is shown. Initial trees for the heuristic search were obtained by applying the Neighbor-Joining-Interchange (NNI) method to a matrix of pairwise distances estimated using a JTT model. To model evolutionary rate differences among sites Gamma distribution (four discrete categories) with Invariant sites (G + I) was used. All positions with <95% site coverage were eliminated, i.e., <5% alignment gaps, missing data and ambiguous amino acids were allowed at any position. 500 bootstraps were done to assess the robustness of the phylogeny.

### Identification of CWDEs

Genes coding for enzymes with carbohydrate substrates were identified using the CAZy database [51, 52]. CWDEs belong to CAZy families GH, CE, PL and AA. Therefore, all members of these families were subjected to secretion analysis by using the SecretomeP algorithm [55]. A neural network score of ≥0.6 was taken to predict secreted CAZymes, i.e. CWDEs.

### Identification of gene clusters

To predict putative secondary metabolism gene clusters *in silico* two different algorithms were used, the Secondary Metabolite Unique Regions Finder (SMURF, [153]) and the Motif Density Method (MDM, [154]). With SMURF predictions are based on PFAM and TIGRFAM domain content and chromosomal position of a gene. MDM uses the density of occurrences of cluster-specific regulatory motifs in the promoter regions as an additional criterion to distinguish cluster from non-cluster genes. Application of the two algorithms to the *Rhynchosporium* BCG genomes yielded putative *PKS* gene clusters with varying degrees of overlap.

### Identification of effector candidates

Candidate effector genes were identified by screening the genome for gene models whose products fulfill the criteria (1) secretion, (2) small size, (3) high cysteine content [117]. The TargetP 1.1 [155] and WoLF PSort [156] protein localization algorithms (NN-based SP score >0.5, prediction confidence from RC = 1 to RC = 3) and transmembrane helix prediction (TMHMM 2.0, TMH = 0; [157] were used to predict secreted proteins. Sorting of the candidates obtained according to length and cysteine content was done to yield proteins with ≤ 200 amino acids and ≥ 2% cysteine residues. The nucleotide sequences of these genes were used as queries in a BLASTn analysis to identify candidates, for which homologous sequences do not exist in the genomes of the other species. In addition, OrthoMCL analysis [118] was carried out on the three BCG genomes for orthology detection. Those proteins that lack any orthologs and paralogs were compared to the ≤200-amino acid protein fraction. Finally, using the single-gene sequences coding for small, cysteine-rich proteins as a query, BLASTn search of the other genomes was done to identify genes that are unique for a given genome.

### EST libraries

Three EST libraries were generated from *R. commune* mycelia, from germinated conidia and from epidermal strips of inoculated barley leaves. Mycelia of isolate UK7 were grown for 14 d in liquid culture, subsequently filtered, air-dried and ground to a fine powder using glass beads (acid washed, 150–212 μm, SIGMA) to efficiently disrupt fungal hyphae [30]. Total RNA was extracted from mycelia using the Trizol method [158], then treated with DNAse (Roche), quality-controlled on a 1.2% agarose gel and shipped for cDNA synthesis using the SMART technology [159]; Evrogen Technologies, Moscow, Russia). After normalization using the duplex-specific nuclease technology [160] the cDNA was 454-sequenced, assembled and trimmed (GATC Biotech, Konstanz, Germany).

Conidia of isolate 214 were incubated in sterile distilled water for 24 h and mRNA was extracted from germinated conidia using Dynabeads Oligo (dT)25 (Invitrogen Dynal AS, Oslo, Norway). RNA yield was measured using a NanoDrop Micro Photometer (NanoDrop Technologies, Inc., Rockland, USA). Prior to cDNA synthesis, RNA samples were treated with DNaseI using the Ambion DNA-free kit following the manufacturer’s protocol. First strand cDNA for sequencing was synthesized from 0.5 μg of mRNA and PCR-amplified for 11 cycles using the SMART PCR cDNA synthesis kit (Clontech Laboratories/Takara Bio Europe SAS, Saint-Germain-en-Laye, France) following the manufacturer’s protocol. The non-normalized cDNA library was 454-sequenced, trimmed and assembled at the University of Liverpool, UK.

Barley plants were inoculated with spores of UK isolate 2LA. At 3 dpi epidermal strips were prepared and total RNA was extracted using an RNeasy Plant Mini Kit (Qiagen, Hilden, Germany) according to the protocol supplied by the manufacturer. After testing RNA integrity by agarose gel electrophoresis, total RNA was sent to the University of Exeter, UK, for mRNA isolation, lllumina sequencing, trimming and assembling. Finally, all ESTs were mapped on the *R. commune* genome.

### Quantitative PCR

Relative fungal biomass and fungal gene expression during pathogenesis were quantified by qPCR with DNA as template and by qRT-PCR with cDNA as template using the efficiency calibrated model [119] as described previously [105, 161]. For qPCR the fungal target gene *GPD* was quantified using the primers GPDRT2s and GPDRT2as, the barley reference gene *TSP* (GenBank accession no. AK359241) using the primer combination TSP_RT_F/TSP_RT_R (Additional file 12: Table S7). For qRT-PCR, expression of *GPD* or *sterol C4-methyl oxidase* served as reference (primer pairs GPDRT2s/GPDRT2as), whereas RcSP expression was quantified using the primer combinations RT-RcSP1f/RT-RcSP1r (*RcSP1*), RT-RcSP2f/RT-RcSP2r (*RcSP2*), RT-RcSP3f/RcSP3r (*RcSP3*), RT-RcSP5f/RcSP5r (*RcSP5*), RT-NIP2.6-UK7f/RT-NIP26-UK7r (*RcSP6*), RT-RcSP7f/RT-RcSP7r (*RcSP7*) and RT-RcSP9f/RT-RcSP9r (*RcSP9*; Additional file 12: Table S7).

### Generation of fungal *RcSP* deletion mutants

The *RcSP* genes were re-sequenced using the primer pairs RcSPn-seq-s/RcSPn-seq-as (LGC Genomics, Berlin; Additional file 12: Table S7). Constructs for the disruption of *RcSP* genes were generated by fusion PCR [161, 162] and deletion mutants were obtained through split-marker recombination by replacing the *RcSP* genes with a *hph* resistance cassette [121]. To this end, 1000 bp of *RcsP* 5′ and 3′ flanking sequence were amplified in a first step using the primer pairs fusion1_s/fusion2_as and fusion3_s/fusion4_as, respectively, (Additional file 12: Table S7) and genomic DNA as template. After amplifying the *hph* cassette with the primers hph_fusion_s and hph_fusion_as and the vector pAN7-1 [163] as template, in the second step the complete deletion construct was generated with the primers fusionA-s and fusionB-as using the 1000-bp flanking sequences and the *hph* cassette as a template. In the third step, the split-marker constructs overlapping in the *hph* gene by 50 bp were generated using the primer pairs fusionA_s/HPH-r and HPH50-f/fusion-as. Fungal transformation and mutant selection was described previously [105, 161, 164]. Successful deletion of the genes *RcSP1, RcSP2, RcSP5 and RcSP6* was analyzed using the primer pairs RcSPn_dt_s/RcSPn_dt_as, whereas deletion of *RcSP3* and *RcSP9* was shown with the primer pairs RcSPn-seq-s/RcSPn-seq-as. Integration of the disruption construct was proven by amplifying both ends using the primer pairs fusion1_s/gpdA_KO-as and trpC_KO_s/fusion4-as with mutant DNA as template.

## Additional files

Additional file 1: Figure S1. The *Leotiomycetes* class of Ascomycetes. The concatenated nucleotide sequences of 18S rDNA, 28S rDNA, ITS region, elongation factor EF1-a and RNA polymerase II subunits RPB1 and RPB2 from *Rhynchosporium* and twelve *Leotiomycetes* species were used to construct the phylogenetic tree. An enlargement of the *Rhynchosporium* subtree is shown in Fig. 1. Most of the species live a saprobic lifestyle (S). Three are plant pathogens (P) causing brown rot blossom blight disease on stone fruit and occasionally on pome fruit trees (*M. laxa*), grey mold disease on many plant species (*B. fuckeliana*) and the *Phacidiopycnis* post-harvest fruit rot of pear (*P. pyri*), respectively. Numerals on the nodes represent the percentages from 500 bootstraps. Scale: number of substitutions per nucleotide (PPTX 50 kb)
Additional file 2: Table S1. SNPs in organism pairs. (XLSX 11 kb)
Additional file 3: Table S2. (A) Core meiotic recombination machinery genes. (B) Genes involved in sexual reproduction in the aspergilli. (XLSX 43 kb)
Additional file 4: Figure S2. CAZyme numbers in different fungal species. GH, glycosyl hydrolases; CE, carbohydrate esterases; PL, pectate lyases; GT, glycosyl transferases; CBM, carbohydrate binding motifs (modified after [50]). (PPTX 72 kb)
Additional file 5: Table S3. CAZymes. (XLSX 24 kb)
Additional file 6: Table S4. (A) Cell wall degrading enzymes. (B) CWDE numbers according to their substrates. (XLSX 72 kb)
Additional file 7: Table S5. Secondary metabolism gene clusters. (XLSX 43 kb)
Additional file 8: Table S6.
*RcSP* homologs (gene IDs). (XLSX 12 kb)
Additional file 9: Figure S3. Growth acceleration of single deletion mutants. Relative biomass of fungal deletion mutants and wild-type isolate UK7 was determined by qPCR at 14 dpi on barley cv. ‘Ingrid’. Wild-type references are associated to the mutants to the right. Bars represent the 95% confidence intervals. *n*-values are given above bars. (PDF 14 kb)
Additional file 10: Figure S4. Growth of DRcSP9 mutants. Relative biomass of three independent deletion mutants and wild-type isolate UK7 were determined by qPCR during pathogenesis on barley cv. ‘Ingrid’. Bars represent 95% confidence intervals. n_mutants_ = 3, n_UK7_ = 4. (PPTX 145 kb)
Additional file 11: Figure S5. Disease phenotype of DRcSP9 mutants. Primary leaves of barley cv. ‘Ingrid’ were inoculated with spores of wild-type isolate UK7 or of the mutants and photos were taken at indicated times post inoculation. C, mock inoculation. (PPTX 382 kb)
Additional file 12: Table S7. Primers used in this study. (DOCX 17 kb)
Additional file 13: Table S8. Genbank and internal numbers (*Rhynchosporium*) for taxa used in this study. (XLSX 13 kb)

## Abbreviations

BCG: Beaked conidia group
BLAST: Basic local alignment search tool
CAZyme: Carbohydrate-active enzyme
CBM: Carbohydrate-binding motif
CCG: Cylindrical conidia group
CEGMA: Core Eukaryotic genes mapping approach
CWDE: Cell wall degrading enzymes
DMATS: Dimethylallyl tryptophane synthase
EAS: *Euascomycetes*
EST: Expressed sequence tag
ETF1-α: Eukaryotic translation termination factor 1-α
GPD: Glyceraldehyde-3-phosphate dehydrogenase
MAT: Mating type
NIP: Necrosis inducing protein
NLP: NIP2-like protein
NPSL: NRPS-like protein
NRPS: Non-ribosomal peptide synthetase
PKS: Polyketide synthase
RcSP: *R. commune* secreted protein
ROS: Reactive oxygen species
RPB1/2: RNA polymerase II subunits B1/2
SCPCH: Single-copy protein-coding homolog
TC: Terpene cyclase
