# Supplementary material for: Comparative genomics to explore phylogenetic relationship, cryptic sexual potential and host specificity of Rhynchosporium species on grasses
Source: BMC Genomics. 2016 Nov 22;17:953. doi: 10.1186/s12864-016-3299-5 (PMC5118889; doi:10.1186/s12864-016-3299-5)
Supplement: Additional file 1: Figure S1. — The Leotiomycetes class of Ascomycetes. The concatenated nucleotide sequences of 18S rDNA, 28S rDNA, ITS region, elongation factor EF1-a and RNA polymerase II subunits RPB1 and RPB2 from Rhynchosporium and twelve Leotiomycetes species were used to construct the phylogenetic tree. An enlargement of the Rhynchosporium subtree is shown in Fig. 1. Most of the species live a saprobic lifestyle (S). Three are plant pathogens (P) causing brown rot blossom blight disease on stone fruit and occasionally on pome fruit trees (M. laxa), grey mold disease on many plant species (B. fuckeliana) and the Phacidiopycnis post-harvest fruit rot of pear (P. pyri), respectively. Numerals on the nodes represent the percentages from 500 bootstraps. Scale: number of substitutions per nucleotide (PPTX 50 kb) [file 12864_2016_3299_MOESM1_ESM.pptx]

## Slide 1
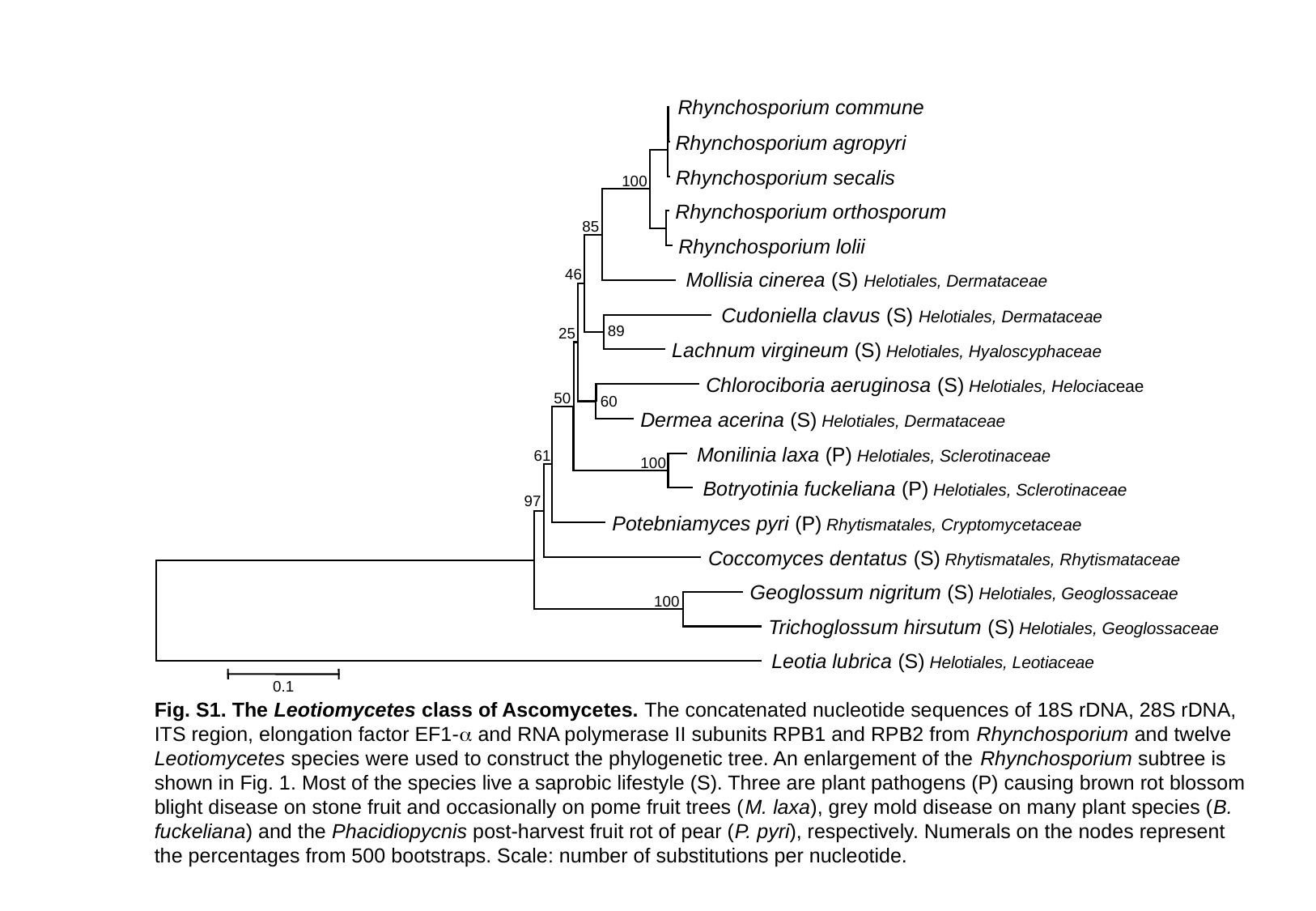

Rhynchosporium commune
 Rhynchosporium agropyri
 Rhynchosporium secalis
100
 Rhynchosporium orthosporum
85
 Rhynchosporium lolii
46
 Mollisia cinerea (S) Helotiales, Dermataceae
 Cudoniella clavus (S) Helotiales, Dermataceae
89
25
 Lachnum virgineum (S) Helotiales, Hyaloscyphaceae
 Chlorociboria aeruginosa (S) Helotiales, Helociaceae
50
60
 Dermea acerina (S) Helotiales, Dermataceae
 Monilinia laxa (P) Helotiales, Sclerotinaceae
61
100
 Botryotinia fuckeliana (P) Helotiales, Sclerotinaceae
97
 Potebniamyces pyri (P) Rhytismatales, Cryptomycetaceae
 Coccomyces dentatus (S) Rhytismatales, Rhytismataceae
 Geoglossum nigritum (S) Helotiales, Geoglossaceae
100
 Trichoglossum hirsutum (S) Helotiales, Geoglossaceae
 Leotia lubrica (S) Helotiales, Leotiaceae
0.1
Fig. S1. The Leotiomycetes class of Ascomycetes. The concatenated nucleotide sequences of 18S rDNA, 28S rDNA, ITS region, elongation factor EF1-a and RNA polymerase II subunits RPB1 and RPB2 from Rhynchosporium and twelve Leotiomycetes species were used to construct the phylogenetic tree. An enlargement of the Rhynchosporium subtree is shown in Fig. 1. Most of the species live a saprobic lifestyle (S). Three are plant pathogens (P) causing brown rot blossom blight disease on stone fruit and occasionally on pome fruit trees (M. laxa), grey mold disease on many plant species (B. fuckeliana) and the Phacidiopycnis post-harvest fruit rot of pear (P. pyri), respectively. Numerals on the nodes represent the percentages from 500 bootstraps. Scale: number of substitutions per nucleotide.
