# Supplementary material for: Comparative genomics to explore phylogenetic relationship, cryptic sexual potential and host specificity of Rhynchosporium species on grasses
Source: BMC Genomics. 2016 Nov 22;17:953. doi: 10.1186/s12864-016-3299-5 (PMC5118889; doi:10.1186/s12864-016-3299-5)
Supplement: Additional file 4: Figure S2. — CAZyme numbers in different fungal species. GH, glycosyl hydrolases; CE, carbohydrate esterases; PL, pectate lyases; GT, glycosyl transferases; CBM, carbohydrate binding motifs (modified after [50]). (PPTX 72 kb) [file 12864_2016_3299_MOESM4_ESM.pptx]

## Slide 1
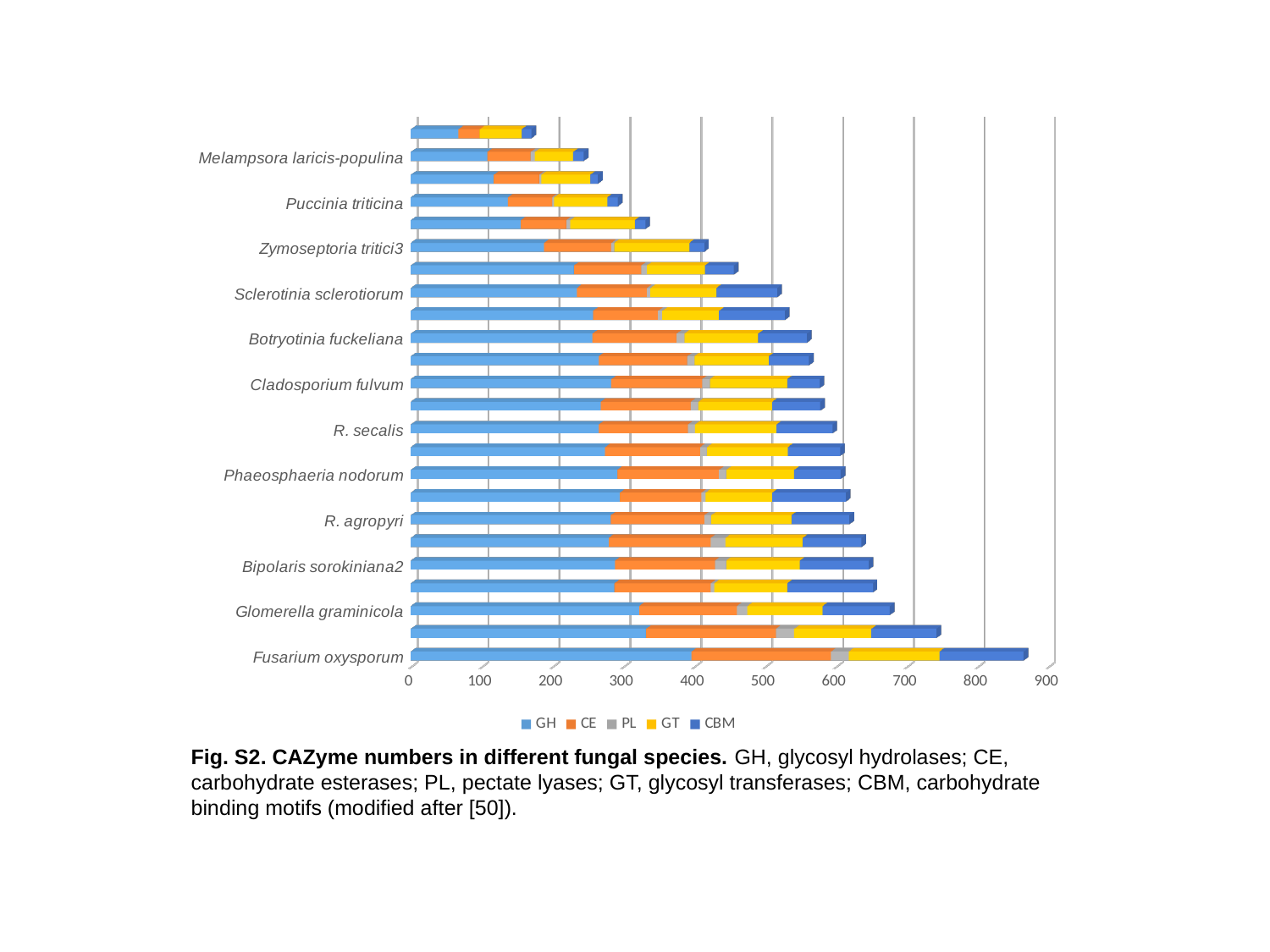

[unsupported chart]
Fig. S2. CAZyme numbers in different fungal species. GH, glycosyl hydrolases; CE, carbohydrate esterases; PL, pectate lyases; GT, glycosyl transferases; CBM, carbohydrate binding motifs (modified after [50]).
