# Supplementary material for: Comparative genomics to explore phylogenetic relationship, cryptic sexual potential and host specificity of Rhynchosporium species on grasses
Source: BMC Genomics. 2016 Nov 22;17:953. doi: 10.1186/s12864-016-3299-5 (PMC5118889; doi:10.1186/s12864-016-3299-5)
Supplement: Additional file 9: Figure S3. — Growth acceleration of single deletion mutants. Relative biomass of fungal deletion mutants and wild-type isolate UK7 was determined by qPCR at 14 dpi on barley cv. ‘Ingrid’. Wild-type references are associated to the mutants to the right. Bars represent the 95% confidence intervals. n-values are given above bars. (PDF 14 kb) [file 12864_2016_3299_MOESM9_ESM.pdf]

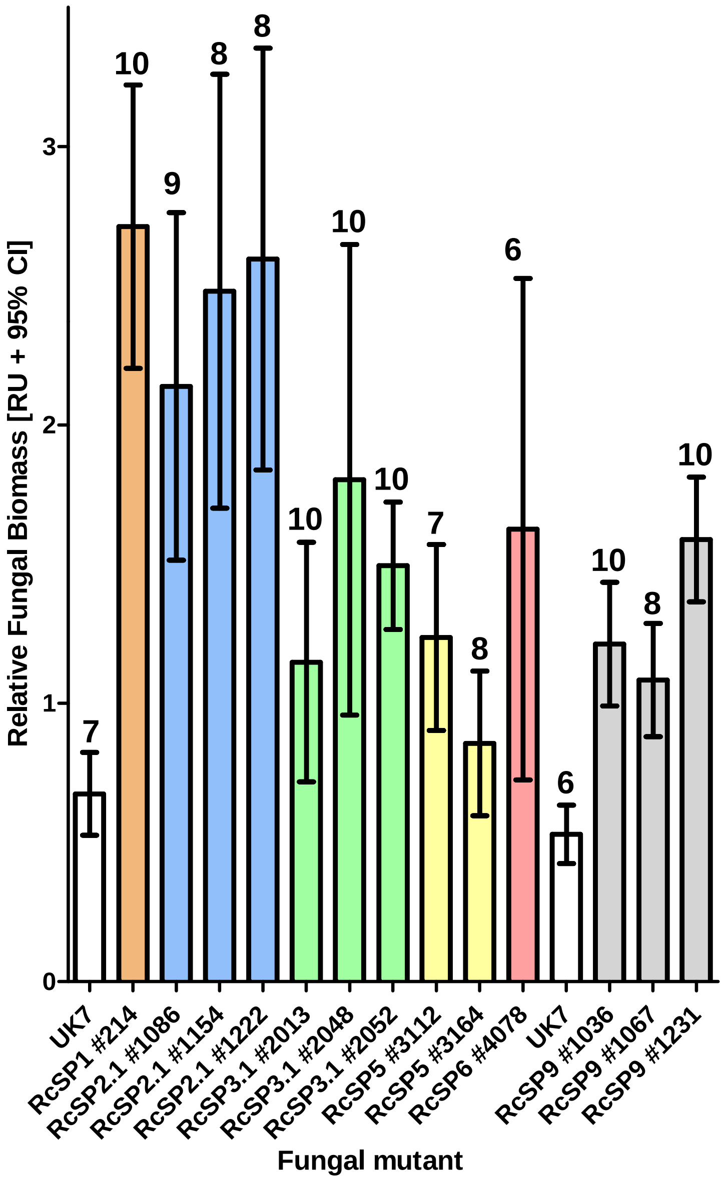

Fig. S3. Growth acceleration of single deletion mutants. Relative biomass of fungal deletion mutants and wild-type isolate UK7 was determined by qPCR at 14 dpi on barley cv. ‘Ingrid’. Wild-type references are associated to the mutants to the right. Bars represent the 95% confidence intervals. N-values are given above bars.
