# Supplementary material for: Comparative genomics to explore phylogenetic relationship, cryptic sexual potential and host specificity of Rhynchosporium species on grasses
Source: BMC Genomics. 2016 Nov 22;17:953. doi: 10.1186/s12864-016-3299-5 (PMC5118889; doi:10.1186/s12864-016-3299-5)
Supplement: Additional file 10: Figure S4. — Growth of DRcSP9 mutants. Relative biomass of three independent deletion mutants and wild-type isolate UK7 were determined by qPCR during pathogenesis on barley cv. ‘Ingrid’. Bars represent 95% confidence intervals. nmutants = 3, nUK7 = 4. (PPTX 145 kb) [file 12864_2016_3299_MOESM10_ESM.pptx]

## Slide 1
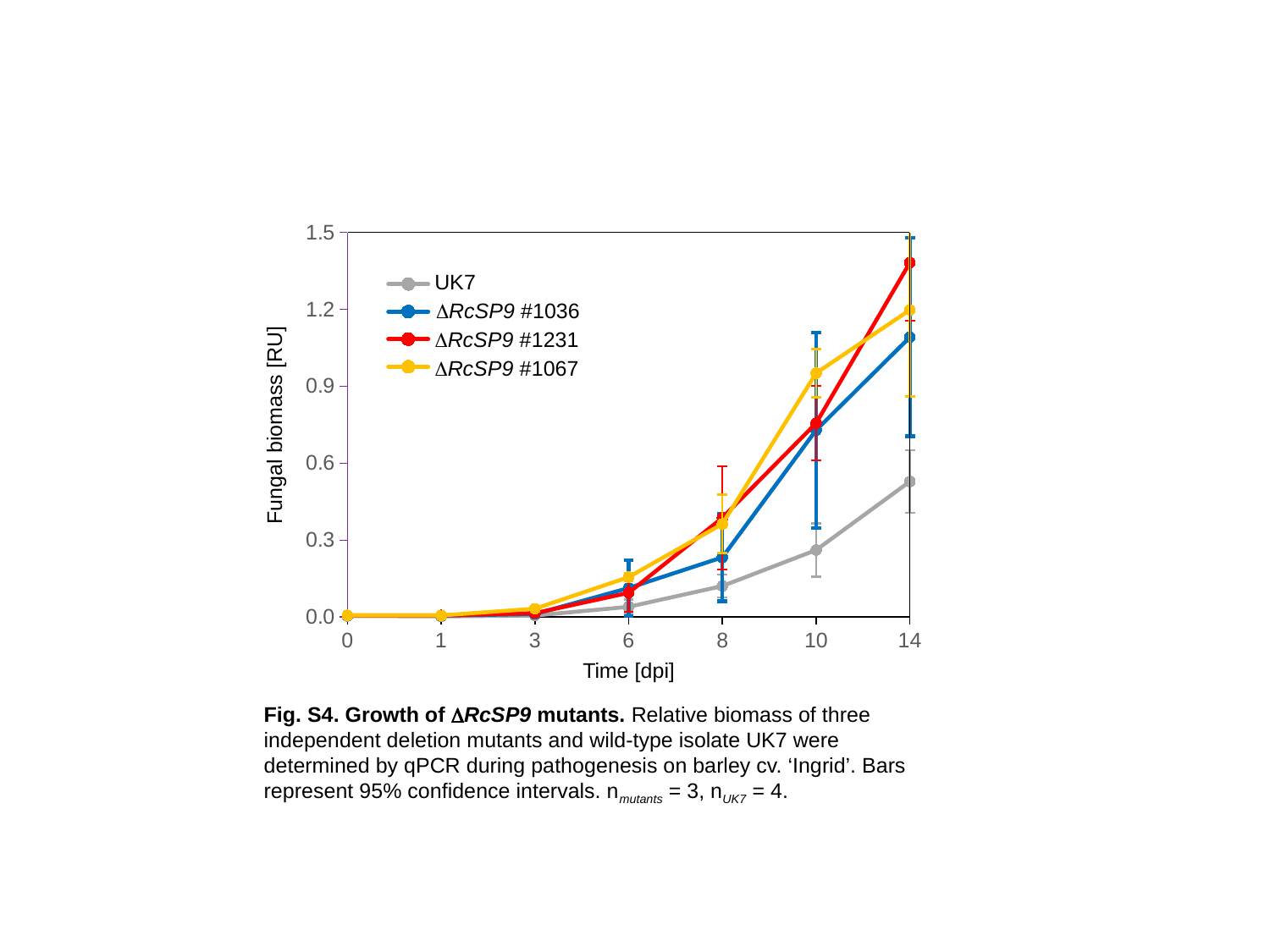

### Chart
| Category | UK7 | DRcSP9 #1036 | DRcSP9 #1231 | DRcSP9 #1067 |
|---|---|---|---|---|
| 0 | 0.003384975139433513 | 0.004727423305202433 | 0.005494976636503381 | 0.005738879969483451 |
| 1 | 0.001936072168849087 | 0.004493155260431034 | 0.005738646811871709 | 0.005607842308646454 |
| 3 | 0.005206153580986651 | 0.010950228591450409 | 0.0143563921043163 | 0.031709356952473955 |
| 6 | 0.03916767632299048 | 0.11233032308536388 | 0.09360918544637592 | 0.15497977166709553 |
| 8 | 0.12019435893982033 | 0.23208967144830897 | 0.3864743781555138 | 0.362476370096574 |
| 10 | 0.26059330233914074 | 0.7284303665579476 | 0.7560188547293403 | 0.9511899829534294 |
| 14 | 0.528199867892812 | 1.091465928586756 | 1.38251196083787 | 1.1977767298176871 |UK7
DRcSP9 #1036
DRcSP9 #1231
DRcSP9 #1067
Fungal biomass [RU]
Time [dpi]
Fig. S4. Growth of DRcSP9 mutants. Relative biomass of three independent deletion mutants and wild-type isolate UK7 were determined by qPCR during pathogenesis on barley cv. ‘Ingrid’. Bars represent 95% confidence intervals. nmutants = 3, nUK7 = 4.
