# Supplementary material for: Comparative genomics to explore phylogenetic relationship, cryptic sexual potential and host specificity of Rhynchosporium species on grasses
Source: BMC Genomics. 2016 Nov 22;17:953. doi: 10.1186/s12864-016-3299-5 (PMC5118889; doi:10.1186/s12864-016-3299-5)
Supplement: Additional file 11: Figure S5. — Disease phenotype of DRcSP9 mutants. Primary leaves of barley cv. ‘Ingrid’ were inoculated with spores of wild-type isolate UK7 or of the mutants and photos were taken at indicated times post inoculation. C, mock inoculation. (PPTX 382 kb) [file 12864_2016_3299_MOESM11_ESM.pptx]

## Slide 1
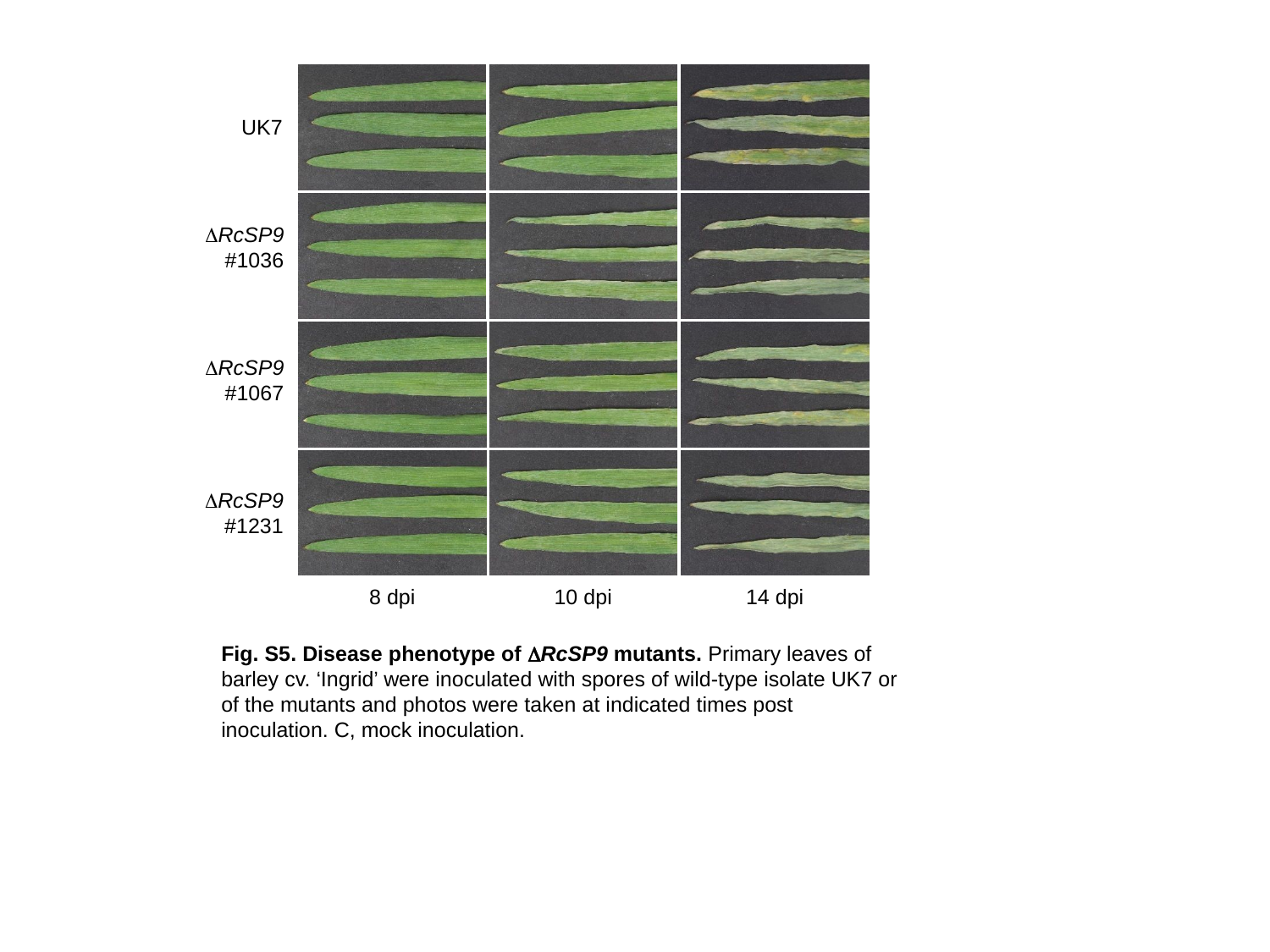

UK7
DRcSP9
#1036
DRcSP9
#1067
DRcSP9
#1231
8 dpi
10 dpi
14 dpi
Fig. S5. Disease phenotype of DRcSP9 mutants. Primary leaves of barley cv. ‘Ingrid’ were inoculated with spores of wild-type isolate UK7 or of the mutants and photos were taken at indicated times post inoculation. C, mock inoculation.
