# Supplementary material for: Comparative genomics to explore phylogenetic relationship, cryptic sexual potential and host specificity of Rhynchosporium species on grasses
Source: BMC Genomics. 2016 Nov 22;17:953. doi: 10.1186/s12864-016-3299-5 (PMC5118889; doi:10.1186/s12864-016-3299-5)
Supplement: Additional file 12: Table S7. — Primers used in this study. (DOCX 17 kb) [file 12864_2016_3299_MOESM12_ESM.docx]

**Table S7. Primers used in this study**

**rDNA sequencing**

18SrDNA1f gtagtcatatgcttgtct

18SrDNA2r tcactgattaatgaaaac

18SrDNA2f tgccaaggatgttttcat

18S-Teil2r acttccatcggcttgagccga

18S-Teil2f aattgcgataacgaacgagacc

18SrDNA3r cttgcatacggtcagcat

18SrDNA3f agtaaagaatgctgaccg

28SrDNAr ccgcttattgatatgcttaa

LROR acccgctgaacttaagc

28SrDNA2r ccaccagagtttcctctggc

28SrDNA2f ggctaggatgctggcgtaat

4_2r tagtctgtgaactgcatccg

4_2f tcaaattgcggggacgtcctg

LR7 tactaccaccaagatct

**qPCR**

GPDRT2s tctgctaaggattggcgcgg

GPDRT2as gcttgccgttgagctctggg

TSP_RT_F gagtccggtcttattgttgagcag

TSP_RT_R gctttcaataacgtcaccctcacg

**qRT-PCR**

GPDRT2as GCT TGC CGT TGA GCT CTG GG

GPDRT2s TCT GCT AAG GAT TGG CGC GG

RT-RcSP1f GCG CCA CCG CAC CCT CTT AT

RT-RcSP1r ATG AAA CCT CCG CCG ATC G

RT-RcSP2f TGC TCA AGG AGG CCC TTT C

RT-RcSP2r GCC GTC CTT AGG ACA GGG T

RT-RcSP3f TGG CCA TAA CTT CGT CGC TTG T

RT-RcSP3r TCT CGT TGT GGA CTC TCC CAT CG

RT-RcSP5f GCC GGC TGG GGG ATG ATT T

RT-RcSP5r GTG CGG CGA CGG TCC AAC A

RT-NIP2.6-UK7f TAT CAA GCT GTC GTC TGT ACG

RT-NIP2.6-UK7r TGA CGC CTG TGT ACA AGT G

RT-RcSP7f TTC ATG TTA CCG GAA CTC CTT TT

RT-RcSP7r CTC TTT GAC CAA TGC AAG AGG TA

RT-RcSP9f CGG CCC AAT TTG TAC GCA GAG A

RT-RcSP9r CTG GTG CAC GTT CCA TAA

**Gene deletion**

RcSP1-fusion1_s aataatcactaagtacgcta

RcSP1-fusion2_as ttctgtacctaggttaatggggttgcggtaggttggatct

RcSP1-fusion3_s cactccacatctccactcgagaagggttcttgagagtatc

RcSP1-fusion4_as atagctatagaaagagaata

RcSP1-fusionA_s ggttcgctagtaataatcta

RcSP1-fusionB_as agagactaggatttcgctta

RcSP2-fusion1_s gagatttgctagaactgcta

RcSP2-fusion2_as ttctgtacctaggttaatggtttcgttaacgatgatgaag

RcSP2-fusion3_s cactccacatctccactcgatcaagacattgccctatgga

RcSP2-fusion4_as agtcaacattgaaagccgag

RcSP2-fusionA_s cactcctttacagagtaggg

RcSP2-fusionB_as tgacaaggattctgctcctg

RcSP3-fusion1_s cagcctatgtagacagcatc

RcSP3-fusion2_as ttctgtacctaggttaatggtttgaagaatcaagtgagtt

RcSP3-fusion3_s cactccacatctccactcgattgtattgatatatgtcaaa

RcSP3-fusion4_as aatatggacattgctacaag

RcSP3-fusionA_s gtatgccagctcgctgcgaa

RcSP3-fusionB_as ccttacaatggctttacttc

RcSP5-fusion1_s agctagaagggatggcggat

RcSP5-fusion2_as ttctgtacctaggttaatggctttgcaagtgtcaggcaat

RcSP5-fusion3_s cactccacatctccactcgaaggaggagagaaattacaga

RcSP5-fusion4_as ccacaatttcgtgtatagac

RcSP5-fusionA_s tttcaatctcgagaacctcc

RcSP5-fusionB_as gtcgcctattcagagtgcat

RcSP6-fusion1_s tgatcgacagtcgtccgatc

RcSP6-fusion2_as ttctgtacctaggttaatggagtgagagtgatagaagtgg

RcSP6-fusion3_s cactccacatctccactcgaacatgacatctggattcaag

RcSP6-fusion4_as gcttttttcggagggggg

RcSP6-fusionA_s cgtacctaattagtccatac

RcSP6-fusionB_as tctccctattagtagaaaca

RcSP9_fusion1_s ctctgtgtactgggcttata

RcSP9_fusion2_as ttctgtacctaggttaatggagtagtaagcttgttgagta

RcSP9_fusion3_s cactccacatctccactcgaaggtgtacaagagagctatt

RcSP9_fusion4_as gagcttcaggcagtgaatta

RcSP9_fusionA_s cttaagcctttgcatcgcaa

RcSP9_fusionB_as gaacttgacttgcttgcttc

hph_fusion_s ccattaacctaggtacagaa

hph_fusion_as cactccacatctccactcga

HPH-r agtccggcacctcgtgcacg

HPH50-f gctgatgctttgggccgagg

**cDNA sequencing**

RcSP1-seq-s atgaagcttggcctcctttc

RcSP1-seq-as ctaatccttcctggcaaccc

RcSP2-seq-s atgaagcccatcaccttatg

RcSP2-seq-as tcaacctattgtatagcg

RcSP3-seq-s* atgcatttctctattcttct

RcSP3-seq-as* ttaagagcatgtaaggtagc

RcSP5-seq-s atgaagctcctcctcctcct

RcSP5-seq-as tcagagatctttattgccaa

RcSP6-seq-s atgaagcatctctctatcct

RcSP6-seq-as tcaacttaggacagcgtact

RcSP9_seq_s* ataagacagctcctgtaccc

RcSP9_seq_as* gtatccaagccagtacaacc

* also used for proof of deletion

**Proof of deletion**

RcSP1_dt_s tcaccagacaccctcttgtt

RcSP1_dt_as ttatctcgcctcctgagaa

RcSP2_dt_s tcaaagccgaggatagagtc

RcSP2_dt_as ttctatcgtcaggttgcga

RcSP5_dt_s ttgatagcgagacaactgac

RcSP5_dt_as ttcatgtcctaagaagcgt

RcSP6_dt_s tccaggcacatcatcctcac

RcSP6_dt_as ttgtccctagcgcagtcgta

**Proof of integration**

gpdA_KO_as gaagtaggtagagcgagtac

trpC_KO_s cagaatgcacaggtacacttg
